# Laminar Flow Alters EV Composition in HUVECs: A Study of Culture Medium Optimization and Molecular Profiling of Vesicle Cargo

**DOI:** 10.1002/smtd.202401841

**Published:** 2025-07-04

**Authors:** Arefeh Kardani, Jan Hemmer, Britta Diesel, Vida Mashayekhi, Annika Schomisch, Marcus Koch, Claudia Fecher‐Trost, Markus R Meyer, Nicole Ludwig, Shusruto Rishik, Andreas Keller, Jessica Hoppstädter, Gregor Fuhrmann, Alexandra K. Kiemer

**Affiliations:** ^1^ Department of Pharmacy Pharmaceutical Biology Campus C2 3 Saarland University 66123 Saarbrücken Germany; ^2^ INM – Leibniz Institute for New Materials Campus D2 2 66123 Saarbrücken Germany; ^3^ Department of Experimental and Clinical Toxicology Institute of Experimental and Clinical Pharmacology and Toxicology Center for Molecular Signaling (PZMS) Saarland University Kirrberger Str., Building 46 66421 Homburg Germany; ^4^ Core Facility Molecular Single Cell and Particle Analysis Medical Faculty Saarland University 66421 Homburg Germany; ^5^ Chair for Clinical Bioinformatics Saarland Informatics Campus Saarland University 66123 Saarbrücken Germany; ^6^ Department of Biology Pharmaceutical Biology Friedrich‐Alexander‐University Erlangen‐Nürnberg Staudtstr. 5 91058 Erlangen Germany; ^7^ FAU NeW – Research Center New Bioactive Compounds Nikolaus‐Fiebiger‐Str. 10 91058 Erlangen Germany; ^8^ PharmaScienceHub (PSH) Saarland University 66123 Saarbrücken Germany; ^9^ Center for Gender‐Specific Biology and Medicine (CGBM) Saarland University 66421 Homburg Germany

**Keywords:** endothelial cells, extracellular vesicles, microRNAs, network analysis, proteomics, shear stress

## Abstract

Endothelial cells (ECs) experience shear stress associated with blood flow. Such shear stress regulates endothelial function by altering cell physiology. Since most cell culture protocols and media compositions are designed for static cultures and experiments with ECs are predominantly conducted under these non‐physiological conditions, a model for culturing ECs under flow conditions is developed, which more closely mimics their physiological environment. This approach also enables the isolation of EVs while minimizing FCS‐derived contaminants. In this study, a comprehensive assessment of how physiologically relevant cultivation conditions influence the vesicle composition and function of ECs is provided. A detailed investigation is conducted for the effect of different cell culture media on morphology and marker expression of human umbilical cord endothelial cells (HUVECs) and EVs, and optimize the conditions to culture ECs under flow, tailoring them specifically to facilitate the efficient isolation of EVs using a hollow‐fiber system model. These EVs are then characterized and compared to those isolated from traditional static culture conditions. Overall, this study presents a model on isolating EC‐derived EVs under conditions that closely mimic physiological environments, and characterization at their proteome, gene expression, and microRNA profile.

## Graphical abstract

Schematic illustration of the current study. A) Multiple cell culture media are used for HUVEC cultures under both static and flow conditions. Cells are analyzed for morphology, endothelial markers, and gene expression. B) EVs are isolated from static and flow HUVEC cultures, and investigated for particle characterization, transcriptome and proteome profiles. Created using BioRender.com.

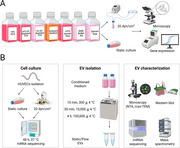

## 1 Introduction

Extacellular vesicles (EVs) are nanosized membrane‐bound structures released by almost all types of cells into their external environment. Eukaryotic EVs are usually classified into three main categories, based on their size and mode of production.^[^ ^1^ ^]^ Microvesicles are formed by the outward budding of membrane vesicles from the cell surface.^[^ ^2^ ^]^ Exosomes originate from the endocytic pathway through the ‘outward’ budding of the late endosomal membrane. Initially, they accumulate in structures known as multivesicular bodies (MVBs), which later fuse with the plasma membrane, releasing their contents as exosomes into the extracellular space.^[^ ^3^ ^]^ The third major type of eukaryotic EVs called apoptotic bodies are produced from cells undergoing programmed cell death by outward budding from the surface of apoptotic cell.^[^ ^4^ ^]^

The significance of EVs was for a long time underestimated, with EVs being initially referred to as cellular ‘dust’.^[^ ^5^, ^6^ ^]^ It is now well recognized that EVs carry a range of bioactive molecules which play a crucial role in intercellular communication, influencing various physiological and pathological processes on their recipient cells.^[^ ^7^, ^8^ ^]^ While originating from the packaging of cytoplasmic contents, EVs are known to harbor numerous proteins, and the presence of these proteins can provide valuable insights into the biogenesis and physiological functions of EVs.^[^ ^9^ ^]^ Moreover, the encapsulated RNAs within vesicles can significantly influence recipient cells by transferring between different cell types. This transformation may manifest as the production of novel proteins in the case of mRNA transfer or the regulation of gene expression with miRNAs.^[^ ^10^ ^]^

EVs produced by human cells are present in various biological fluids, facilitating the delivery of their cargoes not only to neighboring cells within the tissue microenvironment but also over long distances throughout the bodies of multicellular organisms.^[^ ^11^ ^]^ In this context, EVs derived from endothelial cells (ECs), are of particular interest due to their role in vascular homeostasis and their potential as biomarkers for vascular diseases.^[^ ^12^ ^]^ The interaction of endothelial EVs with target cells has various effects on cardiovascular diseases and is dependent on the condition of the donor cells and the molecular cargo within the EVs.^[^ ^13^ ^]^ However, the characteristics and composition of EC‐derived EVs under different physiological conditions remain less studied. Shear stress, a mechanical force exerted by blood flow, plays a crucial role in maintaining endothelial cell function and vascular homeostasis,^[^ ^14^ ^]^ and significantly influences EC behavior, including signaling,^[^ ^15^ ^]^ gene expression,^[^ ^16^ ^]^ and cell morphology.^[^ ^17^ ^]^ This force is critical for maintaining vascular health and can vary across the vasculature in both magnitude and pattern. It is widely recognized that atheroprotective wall shear stress in arteries generally ranges from 10 to 40 dyn cm^−^^2^.^[^ ^18^, ^19^, ^20^, ^21^ ^]^ While previous studies have investigated how shear stress affects EC function,^[^ ^22^ ^]^ its impact on EV characteristics is less explored. Therefore, understanding the effects of shear stress on EC‐derived EVs using experimental models is essential for elucidating the mechanisms underlying vascular health and disease.

Cell culture supernatants are the most used source of EV isolation.^[^ ^23^ ^]^ Fetal calf serum (FCS) is a commonly used supplement in cell culture media as it provides a rich source of nutrients, growth factors, and hormones necessary for cell growth and proliferation. However, its use in EV isolation has been a subject of controversy due to the potential for FCS‐derived components to co‐isolate with EVs and interfere with downstream applications.^[^ ^24^, ^25^ ^]^ To avoid these concerns, several alternatives to FCS‐containing medium have been proposed for EV isolation purposes, including serum‐free and EV‐depleted FCS medium,^[^ ^26^ ^]^ or using supplements like insulin‐transferrin‐selenium (ITS) solution.^[^ ^27^, ^28^ ^]^ However, it is recommended to monitor the changes in cell behaviour and evaluate the background of the analytes of interest to ensure that the chosen method does not affect EV characteristics.^[^ ^29^ ^]^

Here, we identified the optimal media for isolating EVs from primary human umbilical vein endothelial cells (HUVECs), suitable for both static and laminar flow culture conditions. To mimic physiological conditions, we utilized a hollow fiber cartridge to apply laminar shear stress to HUVECs. Additionally, we characterized EVs from static and flow cultures based on morphology, particle size, and content using miRNA sequencing and proteomics approaches. Our results suggest that endothelial EV content differs under the regulation of laminar flow; thus, affecting EV‐mediated mechanisms.

## 2 Experimental Section

### 2.1 Cell Culture

HUVECs were isolated from fresh umbilical cords from female individuals (Klinikum Saarbrücken, Germany, consent of the Local Ethics Committee, permission no. 131/08) under sterile condition using 0.1 g L^−1^ collagenase for digestion (Roche) at 37 °C. To stop the digestion, veins were rinsed with Earle`s medium 199 (PAA, # P04‐07500) containing 10% FCS (#F7524, PAA), 100 U mL^−1^ penicillin G, and 100 µg mL^−1^ streptomycin (#P4333). After centrifugation (10 min, 200 g) cells were resuspended in 5 mL endothelial cell growth medium with supplement mix (# C‐22010, Promocell) containing 10% FCS, 100 U m^−1^ penicillin G, 100 µg m^−1^ streptomycin, and 0.1% kanamycin (#K0254, Sigma), and cultivated at 37 °C and 5% CO2 in a 25 cm^2^ cell culture flask. After one day, cells were washed three times with PBS (phosphate buffered saline, 7.20 g L^−1^ NaCl, 0.43 g L^−1^ KH2PO4, 1.48 g L^−1^ Na2HPO4) and cultivated until confluence. Cells were cryopreserved in passage #1 and used for further experiments.

### 2.2 Laminar Flow

**Figure 1:**
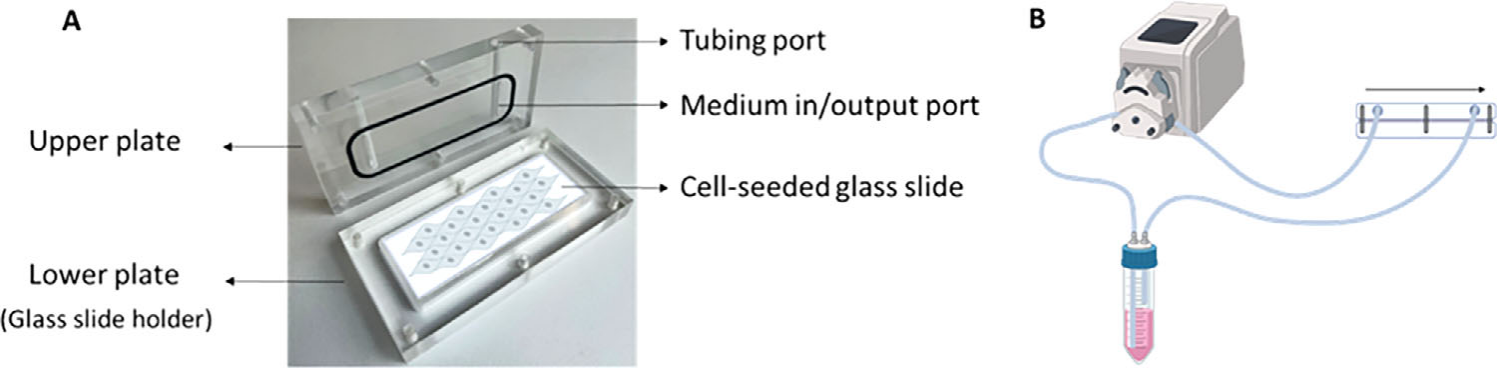
A) One parallel plate flow chamber with cell‐seeded glass slide. B) Schematic illustration of seeded glass slide connected to the peristaltic pump used for morphology, viability, immunofluorescence, and gene expression analysis. Created using BioRender.com.

In this work, two different systems were used to generate laminar flow. A parallel plate flow chamber, which not only provided morphological monitoring of the cells, also was suitable for preliminary experiments in a small scale; and a hollow fiber cartridge for cell culture in a larger scale for EV isolation. Details are mentioned bellow:

To assess morphology, viability, immunofluorescence, and gene expression analysis of flow cultures, the following system was utilized as described previously^[^ ^30^ ^]^ with minor modifications:

Sterilized glass slides (76 × 26 × 1 mm, Roth) were incubated for 30 min in 3 mL collagen (#11179179001, Roche) (50 µg mL^−1^ in 0.2% acetic acid) in 4‐well plates. Then, slides were washed with PBS and after drying for 30 min, cells were seeded onto the glass slides. HUVEC‐seeded slides were incorporated into the parallel plate flow chambers (**Figure** **1**A). The chambers were then linked to a peristaltic pump (403U/VM purple/white, Watson Marlow) and filled with different media (Figure 1B). Laminar flow rates were regulated to fit a shear stress of 20 dynes cm^−2^ and the flow was unidirectional.

The medium flow rate determines the degree of laminar shear stress. To calculate the flow rate (Q) for reaching the shear stress (τ) of 20 dynes cm^−2^, the following formula was used:

(1) $$\tau =\frac{6Q\mu }{bh^{2}}$$

τ = shear stress (dynes cm^−2^), Q = flow rate (cm^3^ s^−1^), μ = viscosity (0.01 dynes s cm^−2^),^[^ ^31^ ^]^ b = channel width (1.9 cm), *h* = channel height (= thickness of the middle part of the chamber (1.15 mm) – thickness of the glass slide).

**Figure 2:**
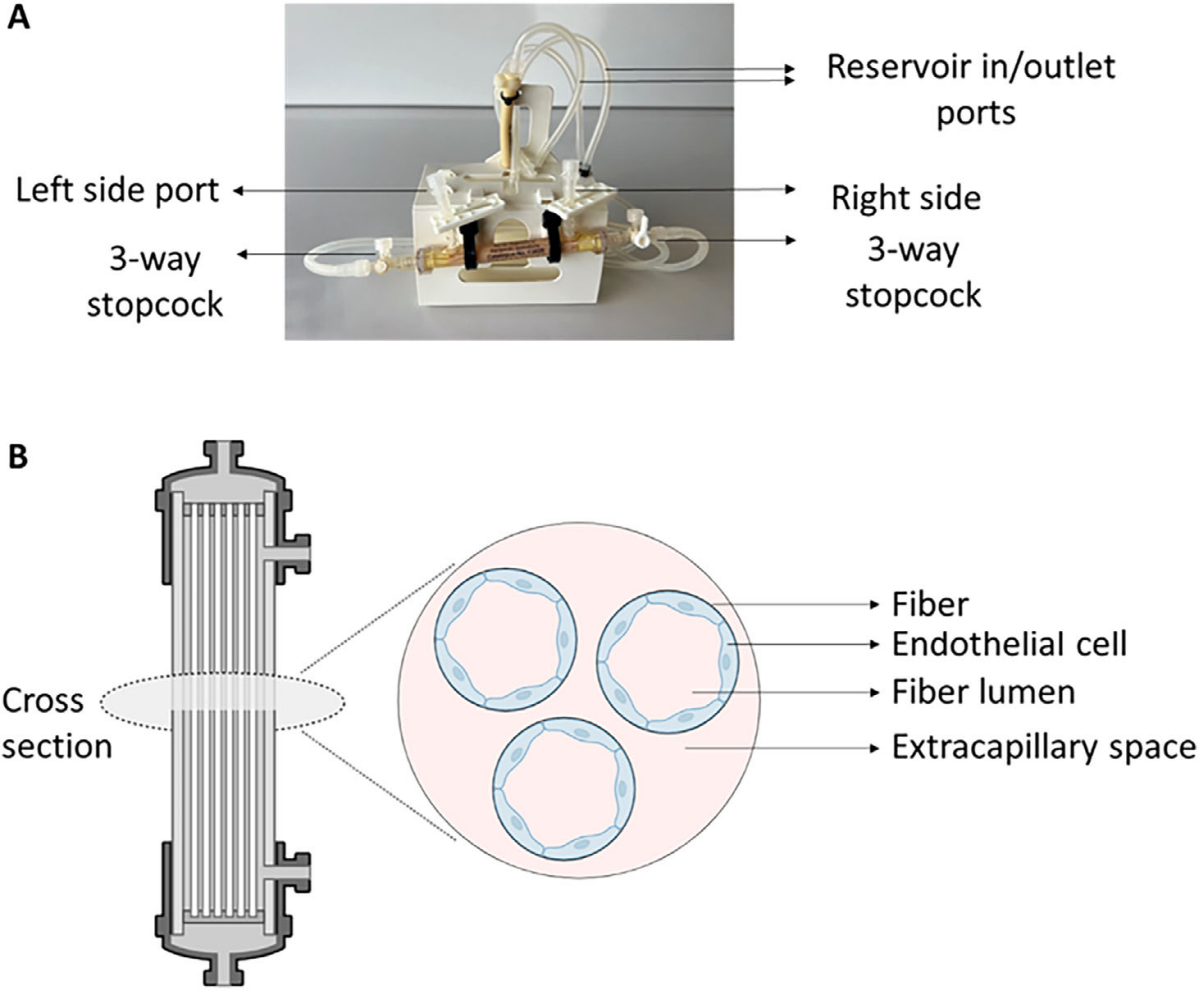
A) One individual cartridge and tubing. B) Schematic illustration of the cartridge and its cross section used to culture HUVECs for EV isolation experiments. Created using BioRender.com.

A hollow fiber cartridge (#C2025, FiberCell system) with the FiberCell Systems Duet Pump^[^ ^32^ ^]^ was used to culture HUVECs for EV isolation experiments (**Figure** **2**).^[^ ^33^ ^]^ Prior to loading the HUVECs into the cartridge, the following preparations were performed according to the manufacturer's instructions.

**1. Activation**: fibers were activated by injecting 70% absolute ethanol using a luer‐lock syringe (#EP97.1, B. Braun, Germany). After ethanol being in contact with the fibers for at least 1 min, excess ethanol was drained, and fibers were rinsed with sterile water.

**2. Coating**: 1 mg mL^−1^ collagen was injected into the fibers (5–10 mL) and incubated for 30 min. Then the fibers were washed by injecting PBS.

**3. Calibration**: complete medium was circulated through the fibers for 1 h at 37 °C with degree 10 on the pump, while the extra capillary space was filled with complete medium as well.

**4. Seeding** was performed according to the manufacturer's instructions.

Laminar flow rates were set to achieve a shear stress of 20 dynes cm^−2^ according to the following formula provided by the manufacturer:

(2) $$\tau =\frac{4Q\eta }{\pi R^{3}}$$

τ = shear stress (dynes cm^−2^)

Q = fluid flow rate (mL s^−1^) (per fiber)

η = viscosity (dyne s cm^−2^)

R = internal radius (0.07 cm)

### 2.3 Morphological Assessment

HUVECs were seeded at 200 000 cells per well in a 6 well plate (2 mL medium per well), and 500 000 cells per sterilized glass slide (76 × 26 × 1 mm, Roth) in a 4 well plate (4 mL medium per well). Cells were incubated overnight to attach. The next day, old medium was removed and replaced with the test medium (**Table** **1**) after PBS wash. Cells were incubated for 72 h under static conditions or under 20 dynes cm^−2^ shear flow. Cells under static culture were imaged with an Incucyte^®^ S3 system every 24 h to monitor morphological changes. Cells under flow condition were imaged with a digital camera (Cannon EODS 400D) attached to a Zeiss AXIOVERT 40 CFL inverted microscope before and after starting the flow.

**Table 1:** Media options.

10% FCS medium (Co)	# C‐22010, Promocell containing 10% FCS (#F7524, PAA)
Endopan medium	# P04‐0065K, PAN‐Biotech
EGM™ BulletKit™ (Lonza)	# CC‐3162, Lonza
ITS solution	# 41400045, Gibco™

### 2.4 Immunofluorescence Staining

HUVEC‐seeded slides were cut with a glass cutter after the incubation time with different media under laminar flow and used for staining. For static culture, 50 000 HUVECs were placed in each well of an 8 well ibidi slide that was coated with 300 µL of 50 µg mL^−1^ collagen. The cells were then incubated overnight before being washed with PBS and exposed to different media for 72 h. Following this, the cells were washed with 300 µl PBS and fixed with 300 µL of 1% warm paraformaldehyde (PFA) for 15 min at room temperature. The cells were then washed again with PBS and permeabilized by incubating for 10 min in 300 µL of 0.1% Triton X‐100. The cells were subsequently washed with PBS and blocked with blocking buffer (#MB‐070, Rockland) for 30 min. 300 µL medium containing antibodies against actin (#P1951, Sigma) and von Willebrand Factor (vWF) (2 µL per well) (#AHP062F, AbD Serotec) was used to stain the cells for 40 min. Excess antibodies were removed by washing the cells with the same blocking buffer; after which the cells were incubated for 10 min with 300 µL of 1µg mL^−1^ Hoechst 33342 (#62249, Thermo Fisher) to stain the nucleus. HCT116 cells were used as negative control. Finally, the cells were observed under a fluorescence microscope (Axio Observer Z1 epifluorescence microscope, Zeiss, Oberkochen, Germany).

### 2.5 Gene Expression

Total RNA was isolated using the Direct‐zolTM RNA MiniPrep Kit (#R2052, Zymo Research). The concentration of isolated RNA was quantified by NanoDrop™ (Thermo Fisher Scientific). Equal amounts of RNA were transcribed using the High‐Capacity cDNA Reverse Transcription Kit (#4368813, Thermo Fisher Scientific) in the presence of an RNase inhibitor (#10777‐019, Invitrogen) according to the manufacturer's instructions. qPCR was performed using a 5xHotFirePol EvaGreen qPCR Mix (#08‐24‐00020, Solis BioDyne) and a total volume of 20 µL. The primer sequences for each transcript are detailed in **Table** **2**. For each primer pair, an annealing temperature of 60 °C was used (except NOS3 with 62 °C annealing temperature). The PCR was performed in a CFX96 touch™Real‐Time PCR detection system (BioRad). Data were normalized to the beta‐actin housekeeping gene (*ACTB*).

**Table 2:** Primer sequences used for qPCR (10 µm stock).

Gene	Accession number	Primer forward sequence	Primer reverse sequence
*ACTB*	NM_001101.3	TGC GTG ACA TTA AGG AGA AG	GTC AGG CAG CTC GTA GCT CT
*NOS3*	NM_00603.4	AACCCCAAGACCTACGTGC	CATGGTAACATCGCCGCAGA
*ICAM1*	NM_000201.3	TGA CCG TGA ATG TGCTCT CC	TCC CTT TTT GGG CCT GTT GT
*KLF2*	NM_016270.2	AGACCACGATCCTCCTTGAC	AAGGCATCACAAGCCTCGAT
*MMP2*	NM_001302510.1	CGTCGCCCATCATCAAGTTC	GAAGGTGTTCAGGTATTGCACTG
*DUSP1/MKP‐1*	NM_004417.4	GGCCATTGACTTCATAGACTCCATC	ACTCAAAGGCCTCGTCCAGC
*HMOX1/HO‐1*	NM_002133.3	GTGCCACCAAGTTCAAGCAG	GCAACTCCTCAAAGAGCTGGA
*VEGFA*	NM_001171623.1	CGCTTACTCTCACCTGCTTCTG	GGTCAACCACTCACACACACAC
*TSC22D3/GILZ*	NM_004089.4	CATGTGGTTTCCGTTAAGCTGG	AGGATCTCCACCTCCTCTCTC
*KLF4*	NM_001314052.2	TGCTCCCATCTTTCTCCACG	TCCCGCCAGCGGTTATTC
*NQO1*	NM_000903.3	CTTGTGATATTCCAGTTCCCCC	GGCAGCGTAAGTGTAAGCAA
*CYP1A1*	NM_000499.5	CATCCCCCACAGCACAACAA	TACAAAGACACAACGCCCCT
*CYP1B1*	NM_000104.4	TCCTCCTCTTCACCAGGTATCC	TGGTCACCCATACAAGGCAG
*PODXL*	NM_005397.4	CCAACAAGCTCGGGACATGA	TAACCGATGACGGTAGGGTG
*NHERF2*	NM_001130012.3	GACCGGCTCATTGAGGTGAA	CGAAGCCGCTTGAAGTGTTC
*ADAMTS1*	NM_006988.5	CACAGCCCATGAATTAGGCCA	ATTGACGCCATCATGTGGGA
*PI16*	NM_153370.3	CTGACAAGCCTAGCGTCGTG	GCTGACCTCTTCACCCTTTG
*CMKLR1*	NM_004072.3	GAGGGGGATCTTGAATGAACAA	GAGGCTGTTGGGGAGACTT
*IGFBP5*	NM_000599.4	ACAAGAGAAAGCAGTGCAAACC	CGTCAACGTACTCCATGCCT
*CCN3*	NM_002514.4	GGCCTTACCCTTGCAGCTTAC	TGCTGTCCACTCTGTGGTCT
*APOLD1*	NM_001130415.2	CGCGGGGACAGAGATGTAAC	GCCTCTCCATTCCCTTTCCAA

### 2.6 Sex Determination of HUVECs

HUVECs were lysed after mixing with 1 µL of Proteinase K (#03115836001, Roche), 5 µL of 10x Taq Buffer (#E00007, Genscript), and 44 µL of water (#A7398, AppliChem) to a total volume of 50 µL. The mixture was then incubated in a heating block set to 55 °C for 60 min at 1500 rpm, followed by 95 °C for 15 min. qPCR was performed as previously described. The primer sequences are detailed in **Table** **3**.

**Table 3:** Primer sequences used for HUVEC sex determination (10 µm stock).

Gene	Accession number	Primer forward sequence	Primer reverse sequence
*RPS4Y1*	NM_001008.4	TTTGCTCATGATTTTGGCACTGT	TCCACAAAAGAATGCCGTCCT
*RPS4X*	NM_001007.5	CAGTGATTAAGTTCTCAGGCAGG	CTTAACAGGGCAGAGGGGTC

### 2.7 EV Isolation

To prepare EV‐depleted FCS, 30% FCS‐containing medium was ultracentrifuged at 100 000 *g* for 18 h at 4 °C, followed by collecting half of the supernatant and filtering through a 0.2 µm stericup filter (Merck Millipore, Germany). The flow‐through was used to prepare 2% EV‐depleted medium. For each biological replicate, 3 individual female HUVECs were mixed when thawing the cryo tube from −80 °C and let grow until confluency. For static culture, cells were seeded into three T75 flasks with 10^6^ cells per flask. The next day, old medium was removed and cells were incubated in 25 mL 2% EV‐depleted FCS medium (Promocell) for 48 h. For flow condition, HUVECs in three T75 flasks were trypsinised and injected (using a luer‐lock syringe) into a collagen‐coated hollow fiber cartridge according to the protocol. Cells were let to attach overnight with the 100 mL^−1^ complete medium flowing through ECS with degree 5 on the duet pump. The next day, the medium in the reservoir bottle was refreshed with complete medium and the direction of flow was connected through the fibers on the cells. The flow was set to 5 overnight. The next day the medium was replaced with fresh medium, and the flow was increased from 5 to 25 degree gradually from morning to afternoon. The cells were incubated for 48 h under laminar flow (20 dynes cm^−2^). After the incubation time, conditioned media were collected and centrifuged for 10 min at 300 *g* at 4 °C to remove remaining cells and debris. The supernatant was subjected for 30 min to 10 000 *g* at 4 °C to remove larger particles. EVs were isolated by ultracentrifuging for 4 h at 100 000 *g* at 4 °C using a 45Ti rotor (Beckman). Due to limitations in EV purification methods, such as sample loss, sample dilution and re‐concentration, the EV pellets were not further purified in this work.

### 2.8 Nanoparticle Tracking Analysis

Particle size distribution and yield of EV preparations were analyzed by nanoparticle tracking analyzer (NTA, LM‐10, Malvern, UK). Preparations of EVs were diluted in 0.22 µm filtered PBS before the analysis. A 500 µLl diluted EV sample was introduced into a green laser‐illuminated chamber to maintain vesicle concentration within the range of 20–120 particles/frame, and a high‐sensitivity video with camera level 13–15 was captured; three videos of 30 s length were recorded and processed by the NanoSight 3.1 software.

### 2.9 Cryo‐TEM Imaging

Cryogenic transmission electron microscopy (cryo‐TEM) was performed on EV pellets after ultracentrifugation. Three to four microliters of the sample were dropped onto a holey carbon grid (type S147‐4, Plano, Wetzlar, Germany) and plotted for 2 s before plunging into liquid ethane at T = −165 °C using a Gatan (Pleasanton, CA, USA) CP3 cryo plunger. The sample was transferred under liquid nitrogen to a Gatan model 914 cryo‐TEM sample holder and analyzed at −173 °C by low‐dose TEM bright‐field imaging using a JEOL (Tokyo, Japan) JEM‐2100 LaB6 at 200 kV accelerating voltage. Images with 1024 × 1024 pixels were acquired using a Gatan Orius SC1000 CCD camera at 2 s binning and 4 s imaging time.

### 2.10 Western Blot

The EV pellets were lysed with Laemmli lysis buffer (50 mm Tris‐HCl, 1% SDS, 10% glycerol, and 0.004% bromophenol blue). HUVECs were also harvested in the same lysis buffer containing 1% protease inhibitors. Samples were boiled for 9 min in 95 °C before loading to the gel. The presence of EV markers was studied by loading equal volumes of samples subjected to 10% sodium dodecyl sulfate‐polyacrylamide gel electrophoresis (SDS‐PAGE) for 20 min at 90 V. Then the voltage was increased to 110 V for another 45 min. Proteins were transferred to polyvinylidene difluoride (PVDF) membrane (#88518, ThermoFisher), under 250 mA for 75 min in 4 °C. Following 1 h incubation in blocking buffer (#MB144 070, Rockland) membranes were probed with primary antibodies for CD9 (1:1000, #MA1‐80307, Thermofischer) and CD63 (1:1000, #sc‐5275, Santa Cruz) overnight at 4 °C. Membranes were washed three times with PBS‐0.05% Tween 20 and incubated in the dark with IRDye 800 CW goat anti‐mouse (1:10 000, Li‐COR Biosciences) for 1 h. The blots were then washed three times for 5 min. Bound antibody was visualized by scanning the membrane with an Odyssey Infrared Imaging System (Li‐COR Biosciences) in 800 nm channel. All blots were cut in order to detect several proteins on the same blot.

### 2.11 Zeta Potential

The surface charge of isolated EVs was measured in triplicates for each batch by DLS using the Zetasizer nano‐ZS (Malvern instruments, Malvern). All samples were diluted 1:500 in 0.22 µm filtered PBS before measurements.

### 2.12 RNA Sequencing

#### 2.12.1 RNA Library Preparation

The library was prepared from static and flow EVs and their parental HUVECs, each in three biological replicates, while each biological replicate was a mix of three individual female donors (EVs from this preparation were used for proteomics as well). RNAs from EVs and cells were isolated using the miRNeasy Serum/Plasma kit (#217184, Qiagen) and Direct‐zol^TM^ RNA MiniPrep Kit (#R2052, Zymo Research) respectively, according to the manufacturer's protocols. RNA concentration was quantified by Nanodrop spectrometer (ThermoFisher Scientific, USA) at 260 nm. Small RNA libraries were prepared according to the MGIEasy small RNA library preparation kit (#1000005269, China). The final small RNA libraries were sequenced by MGI Tech (China).

Libraries for RNA‐Seq were prepared with the MGIEasy rRNA depletion kit and MGIEasy Universal Library Prep Set (MGI Tech, Shenzhen, China) according to manufacturer's protocols. Sequencing was performed on an DNBSEQ‐G400RS instrument by the Sequencing Unit of the Core Facility Molecular Single Cell and Particle Analysis of Saarland University using the 100 bp paired end sequencing strategy.

#### 2.12.2 MiRNA Processing

Fastq sequencing files were analyzed using the miRMaster 2.0 pipeline with default parameters as previously described^[^ ^34^ ^]^ and using miRbase as reference (release 22.1). As an output, miRMaster generated a list with the expression of all mapped miRNAs. We used our in‐house sncRNA pipeline to normalize to rpmmm (reads per million mapped to miRNAs) and filter miRNA based on a raw count detection of at least 5 in > 30% in each group. The normalized count matrices were used to create PCA plots and hierarchical clusterings. Differential expressions were calculated based on t‐tests on the normalized values with multiple testing correction using Benjamini‐Hochberg with a threshold of false discovery rate (FDR) < 0.05 and absolute fold‐change ≥ 2.0. Potential miRNA targets were identified using TargetScan (Release 8.012).^[^ ^35^ ^]^

#### 2.12.3 mRNA Processing

The mRNA module from snakePipes^[^ ^36^ ^]^ was used for processing paired‐end fastq files: STAR^[^ ^37^ ^]^ was used to align to GRChm38 p6 at the gene level, followed by RNA quantification using FeatureCount.^[^ ^38^ ^]^ FastQC^[^ ^39^ ^]^ along with multiQC^[^ ^40^ ^]^ was used for quality checking. The raw count matrix was transformed using Deseq2's^[^ ^41^ ^]^ variance stabilizing transformation and the resulting matrix was used to create PCA plots and gene expression clustering. Fold changes for differential expression was calculated on the raw counts using Deseq2 and a Benjamini‐Hochberg correction and a false‐discovery rate of 0.05 was applied. RPKM values subjected to unsupervised k‐means clustering using iDEP.96. Pathway enrichment analysis was performed using ShinyGO 0.82.^[^ ^42^ ^]^

#### 2.12.4 Integrative Analysis

MiRNA‐mRNA target pairs were obtained from TargetScan Release 8.012.^[^ ^35^ ^]^ Only the pairs with a weighted context++ score about the 75th percentile was kept. Pearson's correlation was calculated using rpmmm values for miRNA and rpkm values for mRNA for matched samples. P‐values were adjusted with the Benjamini‐Hochberg correction with an FDR threshold 0.05.

### 2.13 Proteomics

EVs from three independent preparations were analyzed. 88 micrograms of EV protein were precipitated by trichloroacetic acid (TCA) precipitation with an end concentration of 20% TCA. Samples were washed thrice with acetone. After a final centrifugation of 15 min in a SeedVac Plus concentrator (Savant, Thermo Fisher, Waltham, USA), samples were resuspended in 2x Laemmli buffer (4% SDS, 20% glycerol, 120 mm Tris‐HCl (pH 6.8), 0.02% bromophenol blue in Millipore water) and denatured at 95 °C for 5 min. Proteins were separated on NuPAGE® 4%–12% gradient gels (ThermoFisher Scientific, Karlsruhe, Germany) until the bromophenol dye front reached the center of the gel. Proteins were fixed in the presence of 10% acetic acid /40% ethanol and visualized with colloidal Coomassie stain (10% (v/v) phosphoric acid, 10% (w/v) ammonium sulfate, 20% (v/v) methanol, and 0.12% (w/v) Coomassie G‐250). Six gel pieces were cut/ cell lysate, washed, reduced, carbamidomethylated, and trypsin digested as described before (Fecher‐Trost et al. 2013). After extraction, 6 µl of tryptic peptides were analyzed by data‐dependent nano‐LC‐ESI‐HR‐MS/MS analysis using the instrument setup: Ultimate 3000 RSLC nano system equipped with an Ultimate3000 RS autosampler and Nanospray Flex NG ion source coupled to an Orbitrap Eclipse Tribrid mass spectrometer (Thermo Scientific, Germany). Peptides were separated with a gradient generated with buffer A (water and 0.1% formic acid) and buffer B (90% acetonitrile and 0.% formic acid) at a flow rate of 300 nL min^−1^: 0–5 min 4% B, 5–80 min to 31% B, 80–95 min to 50% B, 95–100 min to 90% B, 100–105 min hold 90% B, 105–106 min to 4% B and 106–120 min to 4% B. Peptides were trapped on a C18 trap column (75 µm × 2 cm, Acclaim PepMap100C18, 3 µm,) and separated on a reverse phase column (nano viper Acclaim PepMap capillary column, C18; 2 µm; 75 µm × 50 cm,). The effluent was sprayed into the mass spectrometer using a coated emitter (PicoTipEmitter, 30 µm, New Objective, Woburn, MA, USA, ionization energy: 2.4 keV). MS^[^ ^1^ ^]^ peptide spectra were acquired using the Orbitrap analyzer (R = 120k, RF lens = 30% m/z = 375‐1500, MaxIT: auto, profile data, intensity threshold of 10^4^). Dynamic exclusion of the 10 most abundant peptides was performed for 60 s. MS^[^ ^2^ ^]^ spectra were collected in the linear ion trap (isolation mode: quadrupole, isolation window: 1.2, activation: HCD, HCD collision energy: 30%, scan rate: fast, data type: centroid).

Peptides and fragments were analyzed using the MASCOT algorithm and TF Proteome Discoverer (PD) 1.4 software (ThermoFisher, Waltham, USA). Therefore, peptides were matched to tandem mass spectra by Mascot version 2.4.0 by searching of a SwissProt database (2021_05, number of protein sequences for all taxonomies: 564.638, for taxonomy human: 20.397). Peptides were analysed with the following mass tolerances: peptide tolerance: 10 ppm, fragment tolerance: 0.7 D. The workflow included tryptic digest and up to two missed cleavage sites. Cysteine carbamidomethylation was set as a fixed modification and deamidation of asparagine and glutamine, acetylation of lysine and N‐term and oxidation of methionine were set as variable modifications. The PD output files were loaded in the software Scaffold (5, Proteome SoftwareInc., Portland, OR, USA). The identification of two unique peptides per protein was set as the minimum for protein identification.

### 2.14 Statistical Analysis

GraphPad Prism 9 software (GraphPad, USA) was used for data analysis. Shapiro‐Wilk test was performed to analyze the data distribution. For normally distributed data, means of two groups were compared with Student's t‐test. For group analysis, one‐way analysis of variance (ANOVA) followed by Dunnett's post hoc test was applied to compare every mean with the mean of control group. All data are presented as mean ± SD, and *p* < 0.05 was considered significant. ^*^ *p* < 0.05, ^**^ *p* < 0.01, ^***^ *p* < 0.001. Schematic illustration were made using BioRender.com.

## 3 Results

### 3.1 Finding the Optimum Medium for EV Isolation

The experiments involving different media were conducted at various times (chronologically), with some options being introduced during later phases of the study. Consequently, not all experiments in this section included all media. **Table** **4** describes the options and the experiments in which they were investigated.

**Table 4:** Media options and experiments.

Options	Morphology	Endothelial characteristics (vWF)	Gene expression	EV production	RNA yield
10% FCS medium (Co)	✓	✓	✓	✓	x
2% EV‐depleted FCS	✓	✓	✓	✓	✓
10% EV‐depleted FCS	✓	✓	✓	✓	✓
Endopan medium	✓	✓	✓	✓	x
EGM™ BulletKit™ (Lonza)	✓	x	x	✓	x
ITS‐supplemented medium	✓	x	x	x	x
FCS‐free medium	✓	x	x	x	x

#### 3.1.1 Cell Morphology

**Figure 3:**
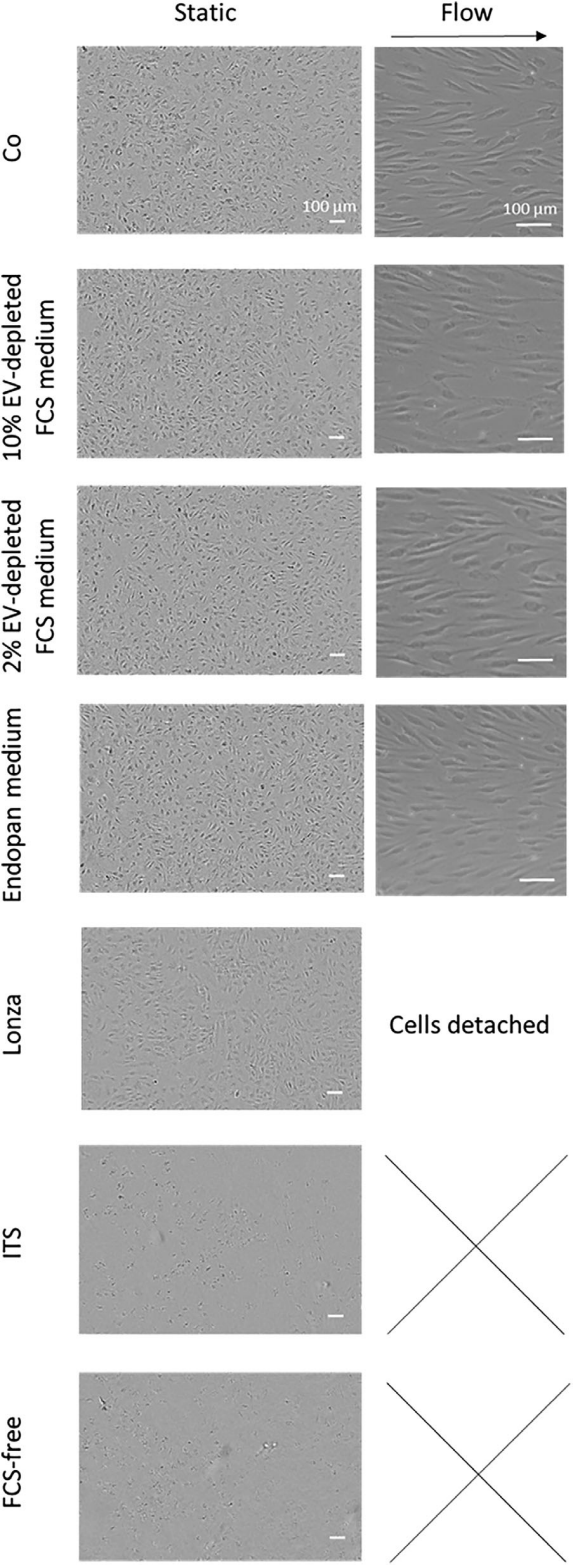
Morphology of HUVECs after 72 h culture in different media under static and 20 dynes cm‐2 flow conditions using the parallel flow chamber. Scale bar = 100 µm. Cells were a mix of two HUVEC donors with unknown sex, conducted in two independent experiments, each including one technical replicate.

The experiment on morphology began by culturing HUVECs under static conditions with the hypothesis that if the cells remained stable in static culture first, then they could be examined under flow. HUVECs were subjected to various media for 72 h, revealing normal morphology in 10% and 2% EV‐depleted FCS medium, Endopan medium, and Lonza medium (**Figure** **3**). However, when grown in ITS‐containing medium and serum‐free medium, some cells were found to be partly detached. Consequently, the first four media were selected to be tested under flow conditions, revealing normal elongation of the cells in the direction of the flow for both EV‐depleted FCS media and Endopan medium, while cells grown in Lonza medium detached under flow.

#### 3.1.2 Von Willebrand Factor

**Figure 4:**
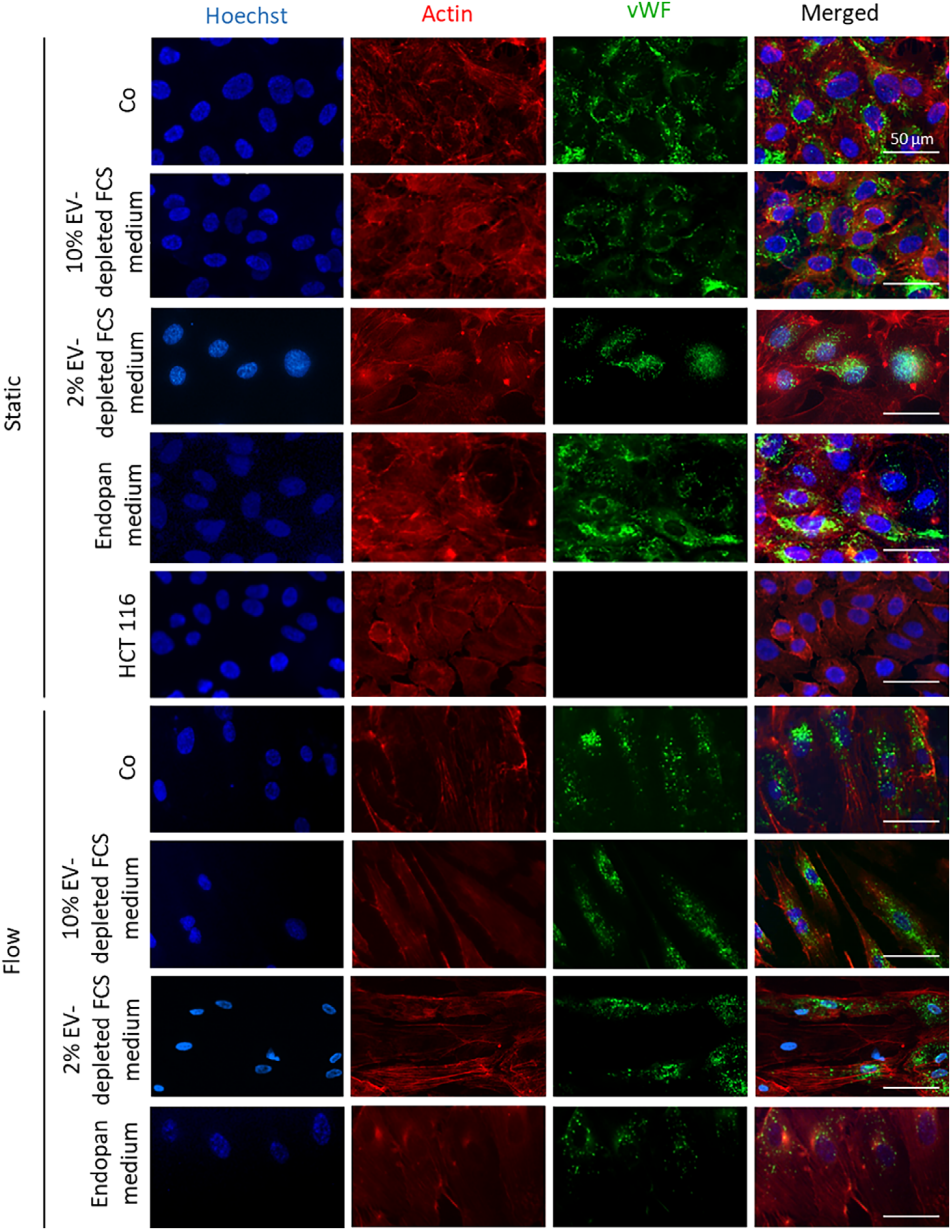
Fluorescence microscopy images of HUVECs cultured under static and laminar flow conditions (20 dynes cm^−2^, using the parallel flow chamber) after 72 h. HCT116 cells were used as negative control. Blue: Hoechst, red: Actin, green: von Willebrand factor. Scale bar = 50 µm. Cells were mix of HUVEC donors, conducted in two experiments, including two technical replicates.

To make sure HUVECs keep their endothelial characteristics, we investigated the presence of von Willebrand Factor as an endothelial marker after incubation with different media under static and flow culture conditions (**Figure** **4**). The immunofluorescent staining detected the presence of vWF in HUVECs cultured in complete (Co), Endopan, 10%, and 2% EV‐depleted FCS medium in both culture conditions.

Quantification of immunofluorescence signal intensities across multiple male and one female donor revealed stable vWF protein expression under both static and flow conditions. In addition, transcriptomic analysis confirmed that vWF mRNA levels remained unchanged under shear stress (data not shown).

#### 3.1.3 Gene Expression

**Figure 5:**
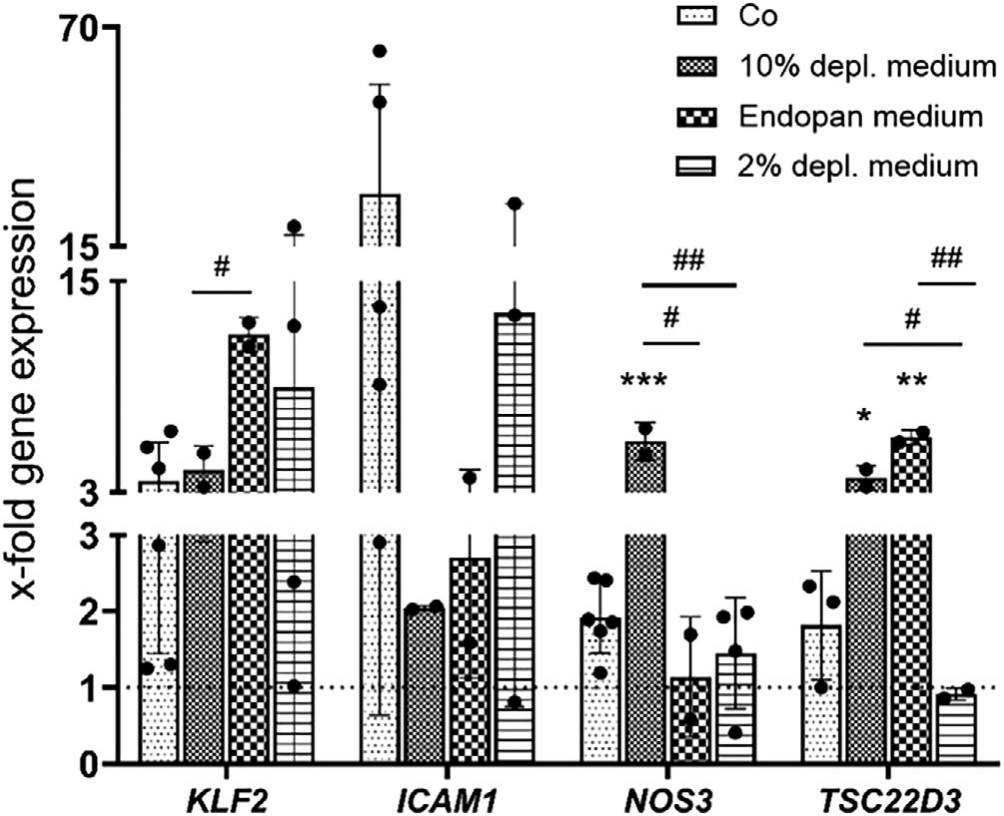
Gene expression of HUVECs incubated with different media under laminar flow conditions using the parallel flow chamber (20 dynes cm^−2^) for 72 h. Data are normalised to static culture as control (dashed line), and shown as mean ± SD. Cells were a mix of two HUVEC donors with unknown sex. Dots show biological replicates, and each dot is the average of three technical replicates. Means of two groups were compared with Student's t‐test. For group analysis, one‐way analysis of variance (ANOVA) followed by Dunnett's post hoc test was applied to compare every mean with the mean of control group. # shows significant differences between groups. * indicates significant differences compared to the control (Co, indicated with the dashed line). *p* < 0.05 is considered significant. ^*^ *p* < 0.05, ^**^ *p* < 0.01, ^***^ *p* < 0.001.

Additional investigations were conducted using qPCR to evaluate the impact of various media on HUVECs on the expression of genes known to be altered upon laminar flow. This aimed to identify a medium that exhibits the least deviation in gene expression compared to the complete medium. Since laminar flow modulates the expression of adhesion molecules and anti‐inflammatory factors,^[^ ^30^, ^43^ ^]^ the expression of relevant genes was examined in HUVECs cultured under laminar flow relative to static cultures. The data indicate a shift in gene expression that closely resembles the control condition when using a medium containing 2% EV‐depleted FCS medium (**Figure** **5**).

#### 3.1.4 RNA Yield

Up until this point, the initial flow culture experiments were conducted using a parallel flow chamber. However, for large‐scale EV isolation, we needed to use a hollow fiber cartridge to culture HUVECs under flow. Before proceeding with large‐scale EV collection, we first needed to optimize the hollow fiber system. Given that the hollow fiber cartridge functions as a closed system, we aimed to ensure cell stability following the incubation period in the 2% EV‐depleted FCS medium that was suggested to be suitable based on microscopic analyses and qPCR data. Attempts to image cells adhered to the fibers using scanning electron microscopy (SEM) were unsuccessful due to limitations in accessing the fibers. Consequently, our alternative approach involved assessing the RNA concentration of the cells. We hypothesized that if the cells remained adherent throughout the incubation period, it should be possible to isolate RNA in a concentration within an acceptable range relative to the initial cell seeding number. The RNA was less concentrated when incubated longer (72 h) in the low serum medium (2% EV‐depleted FCS medium), while the RNA extracted after shorter (48 h) incubation in the same medium had a higher concentration (*n* = 1, cells were a mix of 4 female HUVEC donors) (**Table** **5**).

**Table 5:** RNA concentration of HUVECs.

Medium	Flow incubation time h	RNA C. ng µL^−1^
2% EV‐depleted FCS medium	72	19.5
2% EV‐depleted FCS medium	48	67.5

#### 3.1.5 RNA‐Seq

**Figure 6:**
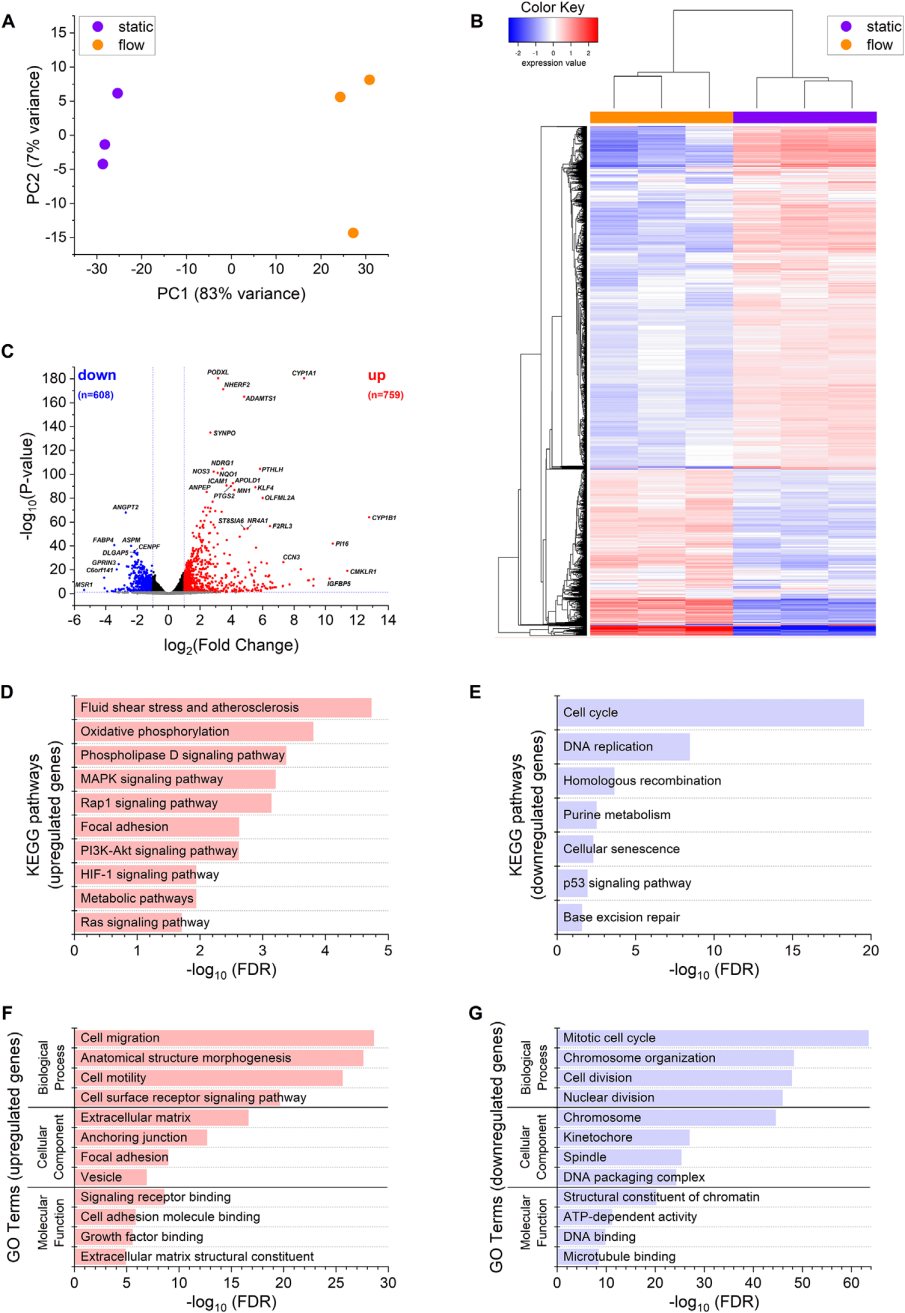
Transcriptomic profiling of female HUVECs under static and flow conditions using the hollow fiber cartridge (*n* = 3). A) Principal component analysis (PCA) of gene expression data. B) Heatmap of the 2000 most variable genes, clustered using hierarchical clustering (Euclidean distance, average linkage). Expression levels are displayed as log10‐transformed RPKM values, with genes centered by subtracting the mean expression across samples. C) Volcano plot of differentially expressed genes (DEGs) between flow and static conditions. Red and blue dots represent significantly upregulated (759) and downregulated (608) genes under flow conditions, respectively (adjusted p‐value < 0.05, fold change > 2). D–G) Pathway enrichment analysis. Selected pathways are shown; see Table S1 (Supporting Information) for the full list. D,E) KEGG pathway enrichment analysis of upregulated D) and downregulated E) DEGs under flow conditions. F,G) GO term enrichment analysis of upregulated F) and downregulated G) DEGs under flow conditions.

Since the 2% EV‐depleted medium suggested to be suitable, we conducted RNA‐Seq with HUVECs under static and flow conditions using the hollow fiber cartridge. Principal component analysis (PCA) revealed distinct clustering patterns driven by culture conditions (**Figure** **6**A). This separation was further confirmed by hierarchical clustering of the 2000 most variable genes, which grouped the samples according to their respective culture conditions (Figure 6B).

Differential gene expression analysis identified 1,367 DEGs (adjusted p‐value < 0.05, fold change > 2), with 759 genes upregulated and 608 downregulated under flow conditions (Figure 6C; Table S1, Supporting Information). Pathway enrichment analysis showed that upregulated genes under flow were mainly involved in mechanotransduction, metabolism, and cellular signaling, with a significant enrichment in the KEGG “Fluid Shear Stress and Atherosclerosis” pathway, confirming that the cells exhibit a well‐characterized endothelial response to flow (Figure 6D; Figure S1, Supporting Information). Conversely, downregulated genes were associated with cell cycle regulation, DNA replication, and repair processes, indicating a shift toward a quiescent endothelial state (Figure 6E).

GO term analysis further supported these findings, with upregulated genes linked to extracellular matrix remodeling, adhesion, and migration, while downregulated genes were enriched in cell division and chromatin organization, reflecting the reduced proliferative activity under shear stress (Figures 6F,G).

**Figure 7:**
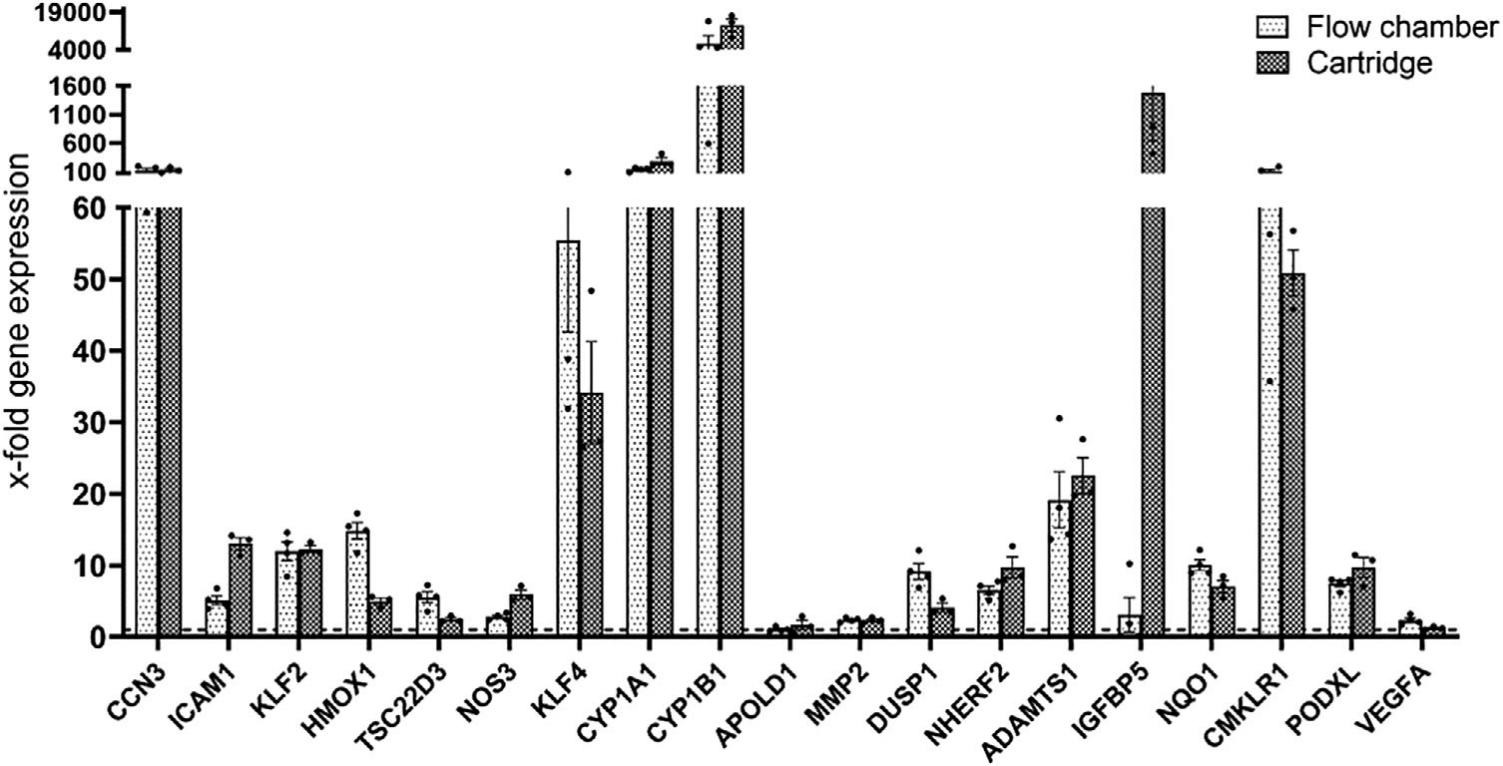
qPCR validation of selected marker genes for laminar shear stress in female HUVECs. Values are shown as x‐fold of static controls (*n* = 3).

To further explore gene expression patterns, we analyzed the 2000 most variable genes using k‐means clustering (Figure S2 and Table S1, Supporting Information), identifying four distinct gene clusters (A–D): Cluster A showed strong upregulation, Cluster B moderate upregulation, Cluster C moderate downregulation, and Cluster D strong downregulation under flow conditions. The functional pathways associated with Clusters A, B, and D closely mirrored those identified in the DEG analysis, reinforcing the observed mechanotransduction and quiescence‐associated signatures. Interestingly, Cluster C was enriched for pathways related to RNA processing, translation, and ribosome function, suggesting a broader suppression of biosynthetic activity. qPCR analysis confirmed the upregulation of shear‐responsive markers in both the parallel flow chamber and hollow fiber cartridge systems, demonstrating consistent gene expression patterns under physiological laminar flow conditions (**Figure** **7**).

#### 3.1.6 EV Surface Markers

**Figure 8:**
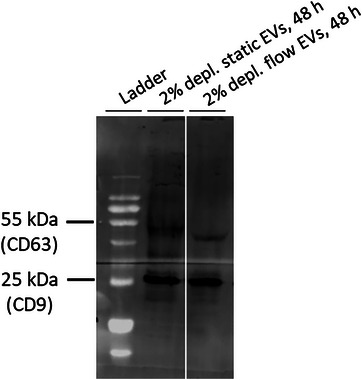
HUVEC EV marker analysis of static and flow cultures after 48 h. Mix of 4 female HUVEC donors was cultured in 2% EV‐depleted FCS medium (under static, and under 20 dynes cm^−2^ laminar flow using the hollow fiber cartridge for 48 h). Presence of EV markers (CD63, CD9) was examined using western blot. 30 µL (30 µg protein) of EVs were loaded into each pocket (*n* = 1).

Since 48 h culture under laminar flow resulted in higher amounts of isolated RNA, we proceeded with large‐scale EV isolation from flow cultures and performed western blot analysis with EVs isolated from conditioned media of cells used for RNA yield analysis to investigate whether lower incubation time would affect EV markers as well. Western blot analysis showed that CD63 and CD9 EV markers are detectable in samples after 48 h under static and flow cultures when 2% EV‐depleted FCS medium is used (**Figure** **8**). These data on EVs obtained from cell culture supernatants after 48 h were in line with surface markers on EVs obtained after 72 h of culture. Interestingly, CD63 was not detectable in serum‐rich media. Given that our data confirmed the suitability of 2% EV‐depleted medium, we did not further investigate this observation (Figure S3, Supporting Information).

Taken together, we decided to culture the cells for 48 h in 2% EV‐depleted FCS medium under static and laminar flow conditions for further EV sample collection and analysis.

### 3.2 HUVEC EV Isolation and Characterization Obtained from Static and Laminar Flow Cultures

#### 3.2.1 EV Characterization

**Figure 9:**
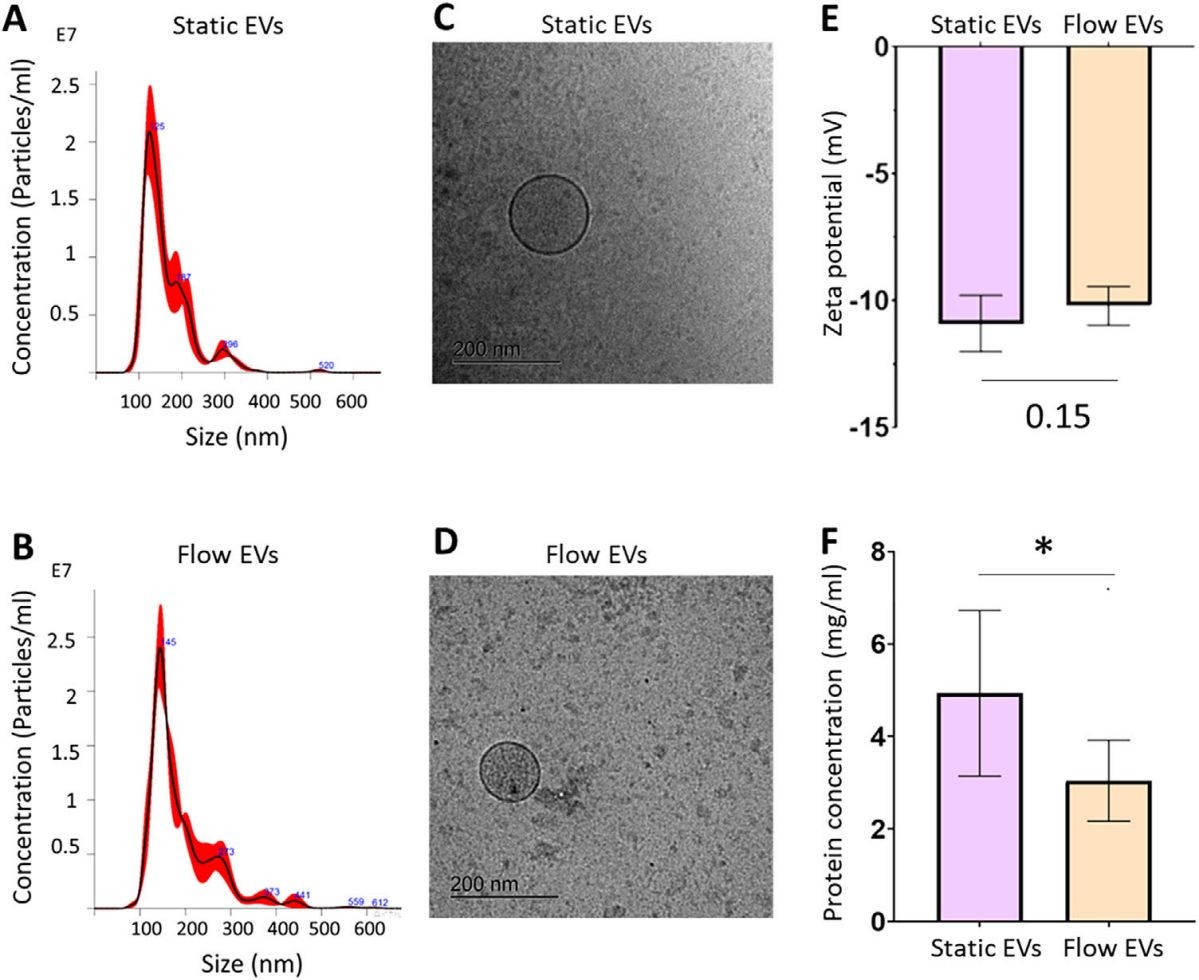
HUVEC EV characterization isolated from static and flow cultures using the hollow fiber cartridge. EVs were isolated using UC from HUVECs cultured in 2% EV‐depleted FCS medium under static and laminar flow conditions (20 dynes cm^−2^) for 48 h. A,B) Representative size distribution of particles by NanoSight particle tracking analysis of static and flow EVs, respectively. C,D) Representative cryo‐TEM images of static and flow EVs, respectively, scale bar = 200 nm. E) Zeta potential of the vesicles (*n* = three biological replicates, each replicate is a mix of three HUVEC female donors). F) Protein concentration of isolated EVs was assessed by BCA assay (*n* = six biological replicates, each replicate was a mix of three HUVEC female donors). Statistical differences were analyzed by Student's t‐test. ^*^ *p* < 0.05.

Having identified the optimal medium for EV isolation suitable for both static and flow conditions, we proceeded with the main EV sample collection of both EV types with three biological replicates (while each biological replicate was a mix of three individual female donors), and their characterization. EVs were isolated by ultracentrifugation from cell culture supernatants from HUVECs cultured in 2% EV‐depleted FCS medium under static and laminar flow conditions (20 dynes cm^−2^) for 48 h. The concentration of EVs was determined using NTA, revealing an average of 2.44 × 10^12^ ± 0.71 × 10^12^ particles per milliliter for static EVs and 2.29 × 10^12^ ± 0.54 × 10^12^ particles per milliliter for flow EVs. Furthermore, NTA showed 129 ± 3 and 134 ± 9 nm for the mode size of static and flow EVs, respectively (**Figure** **9**A,B). The morphology of the EVs was then verified through cryo‐TEM, which confirmed their spherical structure for both EV types (Figure 9C,D). The zeta potential of the vesicles was negative, averaging from −10.9 ± 1.12 mV for static EVs to −10.2 ± 0.77 mV for flow EVs (Figure 9E). The average protein concentration was significantly higher in static EVs (Figure 9F) (**Table** **6**).

**Table 6:** HUVEC EV characterization isolated from static and flow cultures from three individual EV isolations each measured in triplicates.

	Static EVs	Flow EVs
Particle c. (particles per milliliter)	2.44 × 10^12^ ± 0.71 × 10^12^	2.29 × 10^12^ ± 0.54 × 10^12^
Size (nm)	129 ± 3	134 ± 9
Zeta potential (mv)	−10.9 ± 1.12	−10.2 ± 0.77
Protein c. (mg mL^−1^)	4.93 ± 1.8	3.1 ± 0.87

#### 3.2.2 miRNA‐Seq Analysis of Cells and EVs

**Figure 10:**
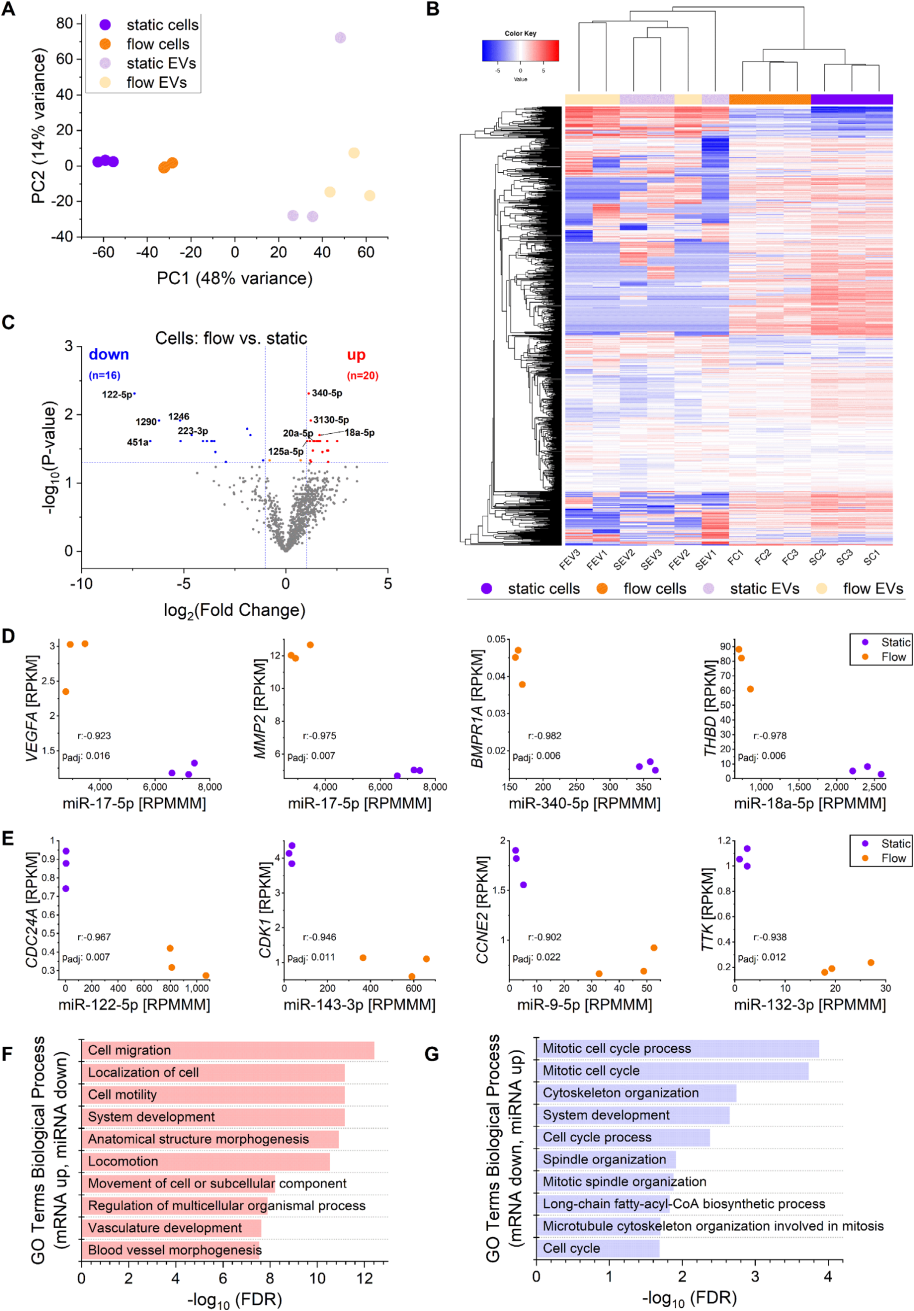
miRNA sequencing analysis of HUVECs and EVs under static and flow conditions using the hollow fiber cartridge (*n* = 3, female donors). A) Principal component analysis (PCA) of miRNA expression data from cells and EVs. B) Heatmap of the 1,000 most variable miRNAs, clustered using hierarchical clustering (Euclidean distance, average linkage). SC: static cells, FC: flow cells, SEV: static EVs, FEV: flow EVs. Numbers indicate biological replicates. Values represent log10 rpmmm values. C) Volcano plot of differentially expressed miRNAs in cells (adjusted p‐value < 0.05, fold change > 2). D,E) Inverse correlation between differentially expressed miRNAs and target mRNAs in selected pathways. Colors indicate condition: violet for static, orange for flow. F,G) GO term enrichment analysis of differentially expressed mRNAs with inverse miRNA regulation.

To investigate the impact of shear stress on miRNA expression, we performed miRNA sequencing on HUVECs and their released EVs under static and flow conditions. Principal component analysis (PCA) revealed a clear separation between static and flow cells, while EVs clustered separately from cells but did not exhibit distinct grouping based on culture conditions (**Figure** **10**A). Hierarchical clustering of the 1,000 most variable miRNAs confirmed this pattern (Figure 10B).

Differential expression analysis identified 36 miRNAs significantly regulated by flow in cells (fold change > 2, adjusted p‐value < 0.05), with 20 miRNAs upregulated and 16 downregulated (Figure 10C; Table S2, Supporting Information). However, in EVs, no significant differences were observed between static and flow conditions (Table S2, Supporting Information), suggesting that flow‐induced miRNA changes occur primarily within cells and are not reflected in the EV cargo.

Correlation analysis revealed 409 miRNA‐mRNA pairs with negative correlation and 388 with positive correlation. Among the negatively correlated interactions, 144 were associated with genes upregulated under flow conditions, while 96 involved downregulated genes (Table S2, Supporting Information). Flow‐downregulated miRNAs correlated with mRNAs involved in the KEGG “Fluid Shear Stress and Atherosclerosis” pathway, while flow‐upregulated miRNAs correlated with genes linked to cell cycle regulation. Examples of such inverse interactions are shown in Figure 10D,E, while the complete dataset is provided in Table S2 (Supporting Information).

As KEGG pathway analysis yielded limited results for predicted miRNA targets, we used GO Biological Process (GOBP) enrichment analysis to highlight functional categories. Upregulated mRNAs (with corresponding downregulated miRNAs under flow) were associated with vascular remodelling and endothelial function, including blood vessel morphogenesis, cell migration, and anatomical structure morphogenesis (Figure 10F). In contrast, downregulated mRNAs (with upregulated miRNAs under flow) were enriched in processes related to cell cycle progression, cytoskeleton organization, and spindle assembly (Figure 10G).

Together, these results indicate that shear stress modulates endothelial miRNA expression, with key miRNAs potentially regulating pathways involved in vascular adaptation and cell cycle control, while EV‐associated miRNA cargo remains unchanged under flow conditions.

#### 3.2.3 Enrichment of Specific miRNAs in EVs Compared to Parental Cells

**Figure 11:**
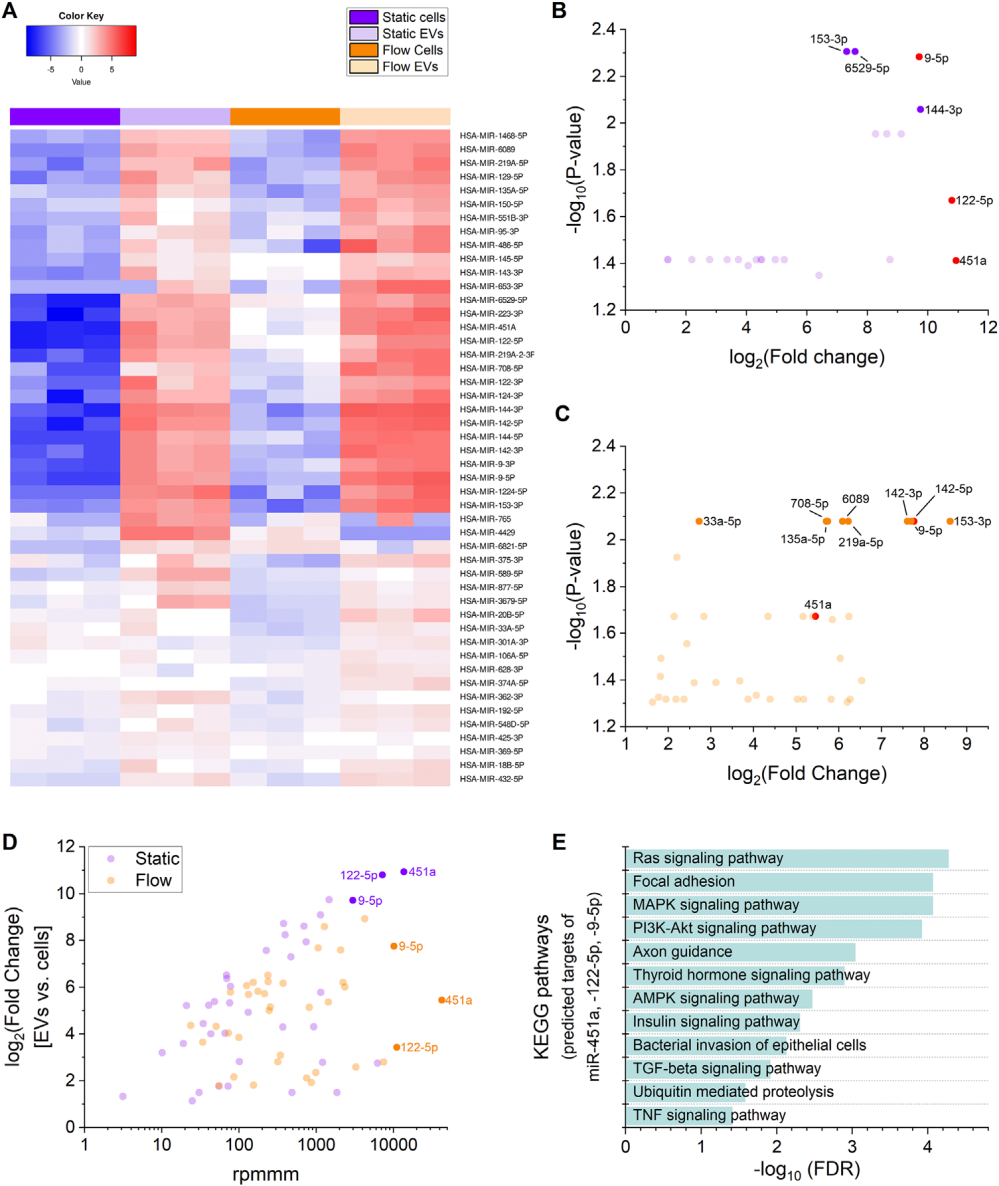
Enrichment of specific miRNAs in EVs compared to parental cells (*n* = 3, female donors). A) Heatmap of miRNAs enriched in EVs compared to their parental cells under static or flow conditions. Displayed are 48 miRNAs that were more abundant in EVs in at least one condition. Values represent log_10_ rpmmm values. B,C) Fold enrichment analysis of miRNAs in EVs compared to cells, plotted against statistical significance (p‐value), for static B) and flow C) conditions. Only miRNAs with fold change > 2 and p < 0.05 are shown. miRNAs with p < 0.01 are highlighted in a darker shade. The most highly abundant miRNAs in EVs (miR‐451a, miR‐122‐5p, miR‐9‐5p, see D) are marked in red. D) Scatter plot of miRNA abundance and enrichment in EVs compared to cells. log₂ fold change (EVs vs. cells) is plotted against rpmmm values for both static and flow conditions. Only miRNAs with fold change > 2 in both conditions are shown. The three most abundant miRNAs in EVs (miR‐451a, miR‐122‐5p, and miR‐9‐5p) are highlighted. E) KEGG pathway enrichment analysis of predicted target genes of the three most abundant EV‐miRNAs.

To identify miRNAs selectively enriched in EVs, we compared miRNA expression levels between EVs and their parental HUVECs under static and flow conditions. A total of 48 miRNAs were more abundant in EVs than in cells under at least one condition (static, flow, or both) (Table S2, Supporting Information). These miRNAs were visualized in a heatmap (**Figure** **11**A).

Fold enrichment analysis confirmed these findings by identifying miRNAs significantly enriched in EVs under static (Figure 11B) and flow (Figure 11C) conditions. While several miRNAs showed strong fold enrichment in EVs compared to their parental cells, miR‐451a, miR‐122‐5p, and miR‐9‐5p stood out due to their exceptionally high absolute abundance in EVs (Figure 11D). These miRNAs were not necessarily the most differentially enriched compared to cells, but their high presence suggests preferential loading into EVs.

To further characterize the potential impact of these highly abundant miRNAs, we performed KEGG pathway enrichment analysis for their predicted target genes. The analysis identified pathways involved in signal transduction, cell adhesion, immune response, and metabolism (Figure 11E; Table S2, Supporting Information). Given that EVs can be taken up by different recipient cells, the functional consequences of these miRNAs may vary depending on the target cell type. For instance, in endothelial cells, these EV‐miRNAs could modulate vascular signaling and barrier integrity, while in immune cells, they may influence inflammatory pathways. Similarly, in metabolic tissues, they could affect energy homeostasis and glucose metabolism. These findings suggest that EV‐associated miRNAs have the potential to fine‐tune diverse cellular processes depending on the microenvironment and recipient cell type.

#### 3.2.4 Proteomics Analysis of Static and Flow EVs

A total of 3268 proteins were detected including 664 proteins unique in static EVs and 520 proteins in flow EVs with 2084 proteins common in both types (**Figure** **12**A; Table S3, Supporting Information). PCA revealed a distinct separation between static and flow EVs, with biological replicates within each category demonstrating similarity (Figure 12B). Fold changes were calculated using the unique spectrum counts of flow EVs/static EVs. Figure 12C illustrates the differentially expressed proteins in a volcano plot, i.e. log_2_ fold change was plotted against −log_10_ p‐value. Negative log_2_ fold change values represent proteins more abundant in static EVs, whereas positive values represent abundant proteins in flow EVs. Cellular component analysis showed that the significantly enriched proteins in both EV types are annotated with exosomal and cytosolic spaces (Figure S4, Supporting Information). Interestingly, within the significant gene ontology (GO) cellular component terms of flow EV proteins, mitochondrial origin was also observed. Next, we analyzed the biological processes that these significant proteins are associated with. Figure 12D shows that the proteins significantly enriched in flow EVs play a role in localization, transport, and respiration. Biological processes associated with enriched proteins in static EVs are shown in Figure 12E, suggesting a role in cellular metabolism, and translation. Based on the mitochondrial origin of flow EV‐enriched proteins as indicated by cellular component terms, and considering their involvement in cellular respiration, proton transport, and energy processes, we conducted a detailed analysis of these proteins. Specifically, we examined their abundance and presence in static EVs as well. Figure 12F illustrates the unique spectrum counts of mitochondrial proteins significantly present in flow EVs, alongside their counts in static EVs. Notably, it demonstrates either an absence or reduced presence of mitochondrial proteins in static EVs. Gene ontology analysis also showed involvement of the mitochondrial proteins in biological processes, such as respiration, oxidative phosphorylation, and ion transport (Figure 12G).

Figure 12Proteomics data of static and flow EVs. A) Number of detected proteins. B) PCA shows a clear distinction between static and flow EVs (*n* = 3). C) Volcano plot representing the differential enrichment between the two EV types. Log_2_ fold change (1.5) is plotted against −log_10_ p‐value (0.05). Top 20 gene ontology (GO) biological processes for proteins significantly enriched in D) flow EVs and E) static EVs according to the STRING database. F) Abundant mitochondrial proteins in static and flow EVs and their distribution. Exclusive unique spectrum count raw data are shown for all three independent preparations per condition (S: static EVs, F: flow EVs). G) Top 20 gene ontology (GO) biological processes for mitochondrial proteins significantly enriched in flow EVs according to the STRING database. N = three biological replicates, each replicate is a mix of three HUVEC female donors.

**Figure:**
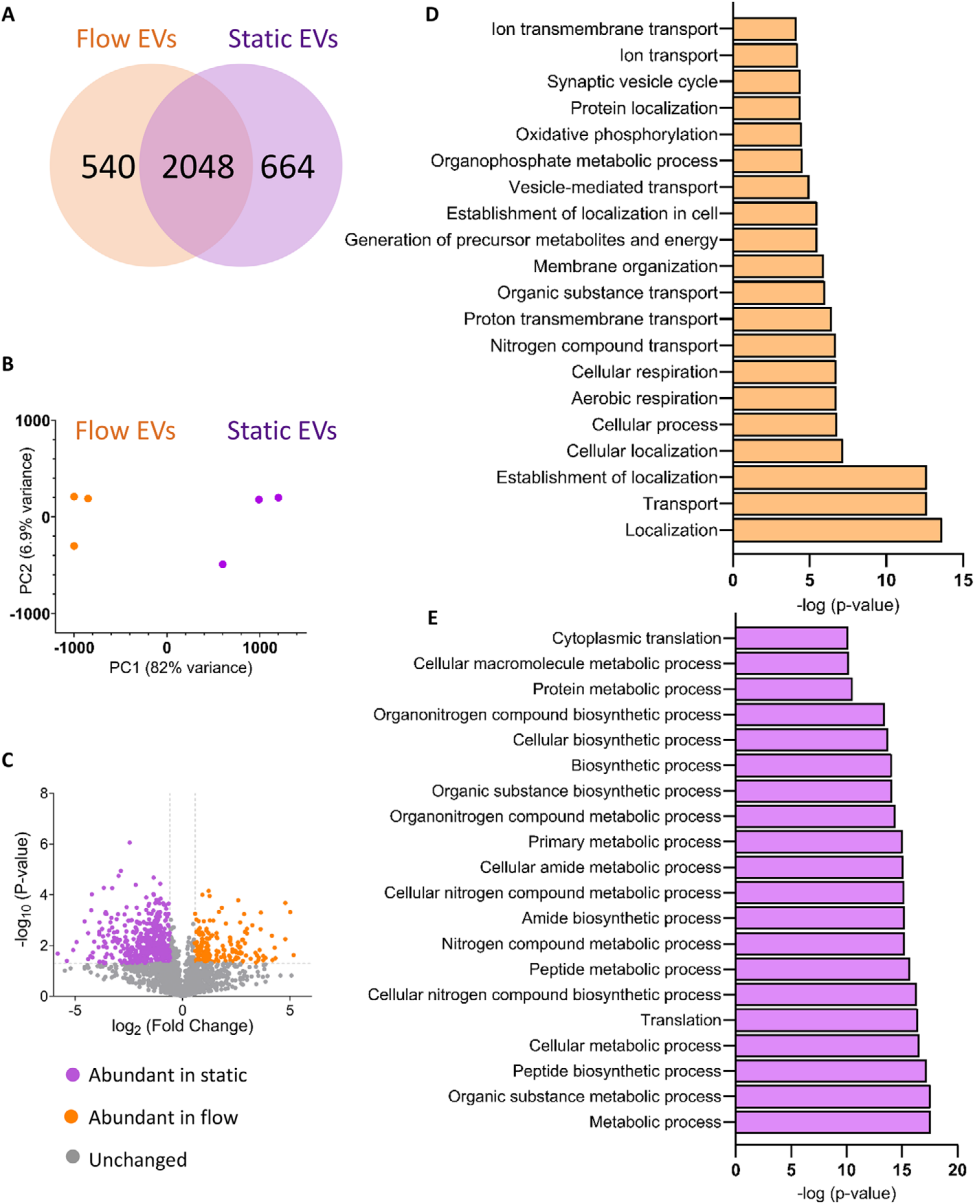

**Figure:**
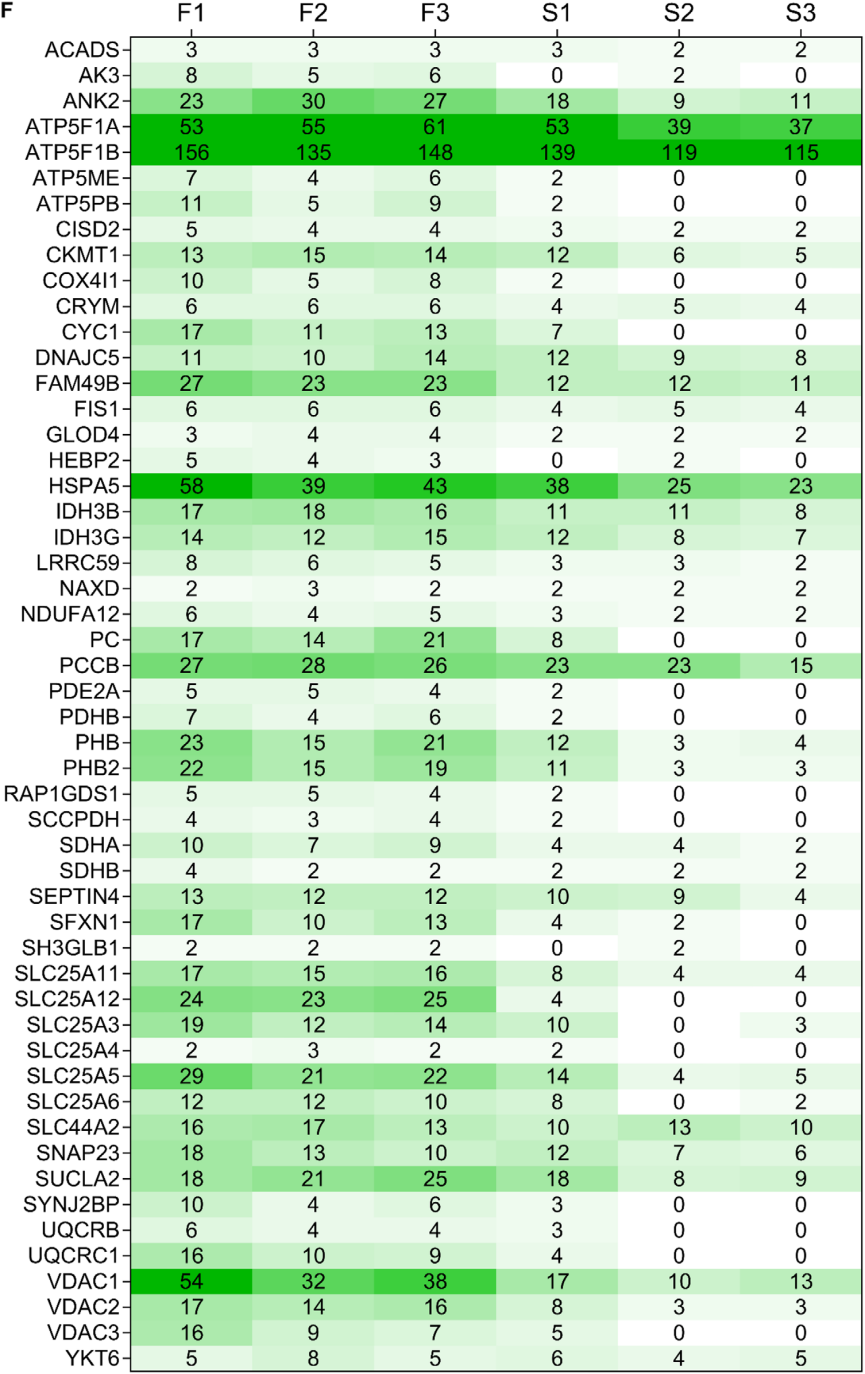

**Figure:**
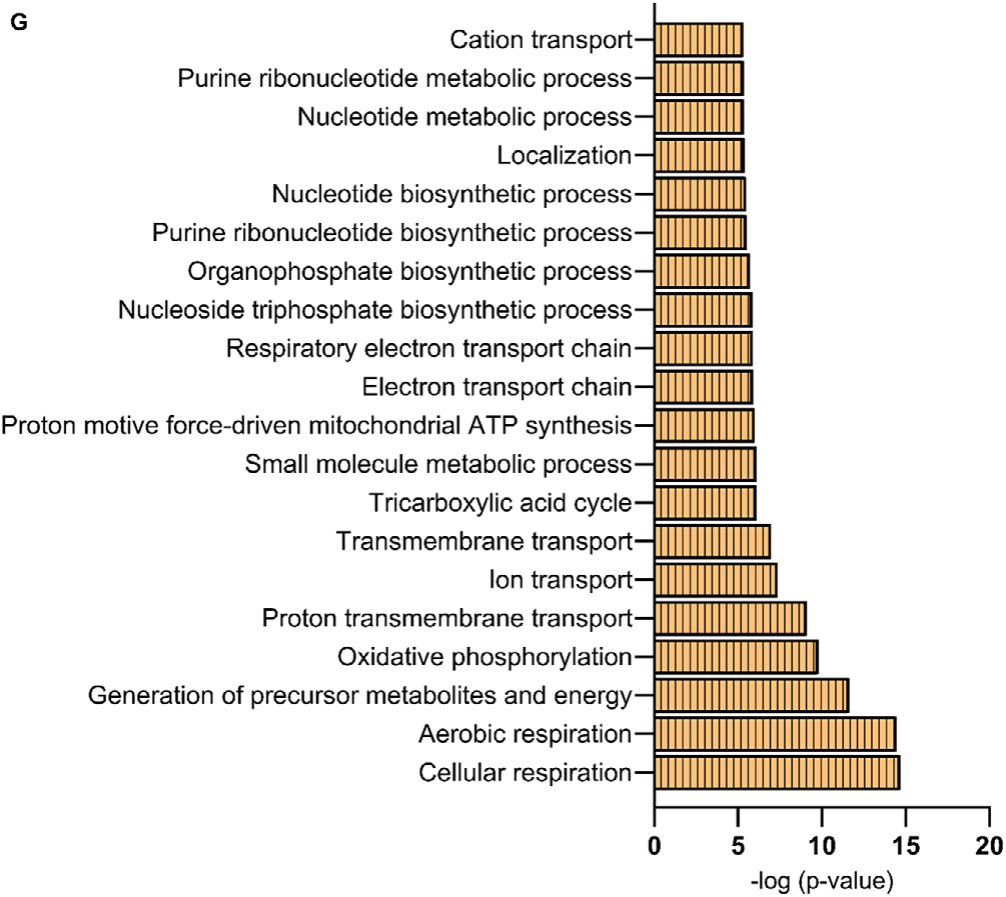

Exclusive, unique spectrum count raw data of a series of EV marker proteins^[^ ^1^, ^44^, ^45^ ^]^ are shown in Figure S5 (Supporting Information) for the independent preparations per condition. Overall, the EV‐specific protein distribution was quite similar in both conditions. Only milk fat globule‐epidermal growth factor‐factor 8 (MFG‐E8) was highly expressed in static EVs compared to the low expression in flow EVs.

## 4 Discussion

In the first step, it was necessary to find an approach to prevent FCS‐derived EV contaminants.^[^ ^29^ ^]^ Although some protocols simply proceed with serum‐free medium for EV isolation from human cell lines,^[^ ^46^, ^47^ ^]^ the utilization of primary endothelial cells in this work, which were intended to be cultured under flow conditions, prevented us from removing FCS from our setting. During the primary setup experiments, these cells were observed to be detached when grown in FCS‐free medium under static culture conditions, leading us to conclude that they would not maintain adherence under the mechanical force of shear flow in serum‐free medium. Consequently, our approach involved an effort to deplete EVs from FCS, aiming to address this critical aspect of our experimental setup.

Shelke et al. compared the centrifugation of FCS for a short (1.5 h) and a long period (18 h) to test the efficiency of these two EV depletion protocols. They found that 18 h centrifugation reduced FCS‐derived EV RNA content by 95%; however, it does not completely eliminate EV contaminants from FCS.^[^ ^48^ ^]^ Later, a study on the effects of serum dilution on the depletion efficiency suggested that the amount of RNA in the EV‐depleted supernatant was reduced in diluted FCS compared to non‐diluted condition, and thus recommended to dilute the FCS to 30% prior to EV depletion.^[^ ^49^ ^]^ Therefore, in this study, a medium containing 30% FCS was ultracentrifuged and then utilized to formulate the primary culture medium for EV production during the incubation period.

One study on the impact of different media on EV production has previously reported that EVs produced from N2a mouse neuroblastoma cells in Opti‐MEM (reduced‐serum medium) were greater in quantity than EVs produced in DMEM‐containing serum.^[^ ^50^ ^]^ Later, the same group attempted to identify specific media components affecting EV production. They found higher levels of EV surface markers (CD9, CD63, and CD81) from HEK293T cells cultured in serum‐free Opti‐MEM compared to serum‐including conditions. Interestingly, a CD81 + EV population was not detectable by western blot analysis when complete medium was used to harvest EVs.^[^ ^51^ ^]^ Also comparing the enrichment levels of genes comprising a certain gene ontology term between the different media conditions, in which cells were cultured for EV production, Bost et al. found that the sphingolipid and ceramide pathways influencing exosome production,^[^ ^52^ ^]^ were upregulated in the Opti‐MEM samples compared to the serum‐containing media. CD63 functions in ESCRT‐independent vesicle formation,^[^ ^53^ ^]^ and ESCRT‐independent exosome formation relies on ceramide generation by neutral sphingomyelinase.^[^ ^54^ ^]^ This could explain the presence of CD63 marker in HUVEC‐derived EVs when low serum amount was employed.

In this work, in addition to static culture condition, we also characterized vesicles isolated from HUVECs subjected to laminar flow trying to simulate the physiological conditions. We utilized two systems to model laminar shear flow. Our data demonstrated strong similarity in gene expression between the two approaches showing upregulation of flow‐induced genes including KLF2—a key master regulator of the shear stress response that governs the expression of ≈70% of flow‐responsive genes.^[^ ^55^ ^]^ These findings validate the effectiveness of both systems in accurately simulating physiological shear conditions. Several fluid shear stress models have been used in the literature. Parallel‐plate flow chambers like the one we used for set up experiments allow the cell layer to be observed with a microscope.^[^ ^17^ ^]^ Cone‐and‐plate systems are used to analyze the shear responses of cells to flow independent of hydrostatic pressure.^[^ ^56^ ^]^ The orbital shaker method is able to generate a larger disturbed flow.^[^ ^57^ ^]^ In recent years, microfluidic systems have been often, allowing the creation of constant or active shear flow with external equipment, like pumps, which dynamically adjust fluid shear stress by altering the inlet flow.^[^ ^58^, ^59^, ^60^ ^]^ However, the choice of a specific model depends on the downstream analysis requirements. Here we used a hollow fiber cartridge system^[^ ^33^ ^]^ that allowed for larger‐scale cell cultivation compared to other commercially available in vitro settings. This made it possible to isolate EVs from a large volume of conditioned medium required for downstream processing; therefore, reducing the number of batches needed for multiple analysis and improving the consistency of the data generated.

Commonly used EV isolation methods including ultracentrifugation, density gradient centrifugation, size exclusion chromatography, and polymer‐based precipitation, vary in EV yield, the depletion of protein contaminants, labour‐intensity, and cost of the procedure. Utilizing a combination of two or more methods has the potential to enhance the removal of protein contaminants; however, it comes at the cost of reducing the overall number of EVs.^[^ ^61^ ^]^ Therefore, the choice of EV isolation method used should depend on the amount of starting material together with the downstream application. Although commercial EV separation kits have been used to isolate EVs from HUVECs,^[^ ^62^, ^63^ ^]^ differential centrifugation has been the most widely used method,^[^ ^62^, ^64^, ^65^, ^66^, ^67^, ^68^, ^69^ ^]^ In our research, we isolated vesicles from the culture medium using ultracentrifugation, without additional purification steps. This decision was due to the noticeable sample loss observed during trial runs of size exclusion chromatography to purify the isolated EVs (Figure S6, Supporting Information).^[^ ^70^ ^]^

Definitive characterization of biogenesis‐based EV subtypes is challenging, as there are no universal molecular markers for ectosomes (also known as microvesicle or microparticle; refers to EVs originating from the cell surface), exosomes (refers to EVs originating from internal compartments of the cell, released via MVBs), or other EV subtypes.^[^ ^71^ ^]^ In our work, we examined a series of EV protein markers based on previous reports,^[^ ^1^, ^44^, ^45^ ^]^ irrespective of the biogenesis routes. A genome‐wide association study for coronary artery disease involving over a million participants identified MFG‐E8 as one of the risk variants and genes associated with cardiovascular diseases,^[^ ^72^ ^]^ positioning it as a potential prognostic biomarker for vascular diseases.^[^ ^73^ ^]^ An in vivo study on endothelial–vascular smooth muscle cell (VSMC) interactions in mice further highlighted the role of MFG‐E8 in driving the pro‐inflammatory phenotypic shift of VSMCs.^[^ ^74^ ^]^ Dysregulated EC‐VSMC communication was shown to potentially contribute to the development of atherosclerosis.^[^ ^75^ ^]^ Among the more abundant proteins in flow EVs, we observed a variety of mitochondrial proteins that were either absent or less prominent in static EVs. This observation aligns with previous reports documenting the presence of mitochondrial proteins in EVs from mouse embryonic fibroblasts and monocyte‐derived dendritic cells.^[^ ^76^, ^77^ ^]^ Vascular endothelial cells sense shear stress generated by flowing blood and transmit this information into the cell interior.^[^ ^78^ ^]^ Previous data have shown a role of mitochondria in the EC mechanotransduction of fluid shear stress.^[^ ^79^, ^80^ ^]^ A recent study suggests that changes in the magnitude and pattern of fluid shear stress alter the mitochondrial content, shape, and intracellular distribution in different vessel regions of a mouse model in vivo and in primary mouse aortic endothelial cells in vitro.^[^ ^81^ ^]^ It has been shown that unidirectional flow induces an elevation of oxidative phosphorylation‐dependent ATP generation.^[^ ^82^, ^83^, ^84^ ^]^ On the other hand, exposing HUVECs to laminar flow (20 dynes cm^−2^) for 24 h decreases glycolysis pathway.^[^ ^85^ ^]^ In line with these findings, we saw an increase in ATP synthase subunits (ATP5MF, ATP5F1A, ATP5F1B, ATP5ME, ATP5PB) and other respiratory chain members (CYC1 (Cytochrome c1. heme protein), UQCRC1 (Cytochrome b‐c1 complex subunit 1), and COX4I1 (Cytochrome c oxidase subunit 4 isoform 1)) in flow EVs. Furthermore, PDP1 (Pyruvate dehydrogenase phosphatase 1), a mediator of glycolysis pathway,^[^ ^86^ ^]^ was not detected in any of the flow EV replicates.

Cellular culture conditions are not only reflected in exosomal proteins but also in miRNA contents. Intracellular miRNA expression profiles of ECs adapt to diverse flow patterns and impact endothelial biology.^[^ ^87^, ^88^, ^89^, ^90^ ^]^ Therefore, we hypothesized that the miRNA content of ECs is also regulated by shear stress. To test this, we performed miRNA sequencing with EVs and cells. A recent study compared extracellular vesicles (EVs) from HUVECs under static and laminar flow conditions using a parallel plate flow chamber with a shear stress of 15 dyn cm^−^^2^ for 8 h.^[^ ^91^ ^]^ In addition to technical differences between this study (employing 10% EV‐depleted FBS) and ours, such as the choice of flow model and flow duration for EV isolation, the authors observed differences in the miRNA profiles of EVs from the two conditions. To validate their flow model, they noted changes in inflammatory gene expression; however, the factors they studied were not among the well‐established flow‐induced transcriptional patterns.^[^ ^16^ ^]^ Although we did not observe significant changes in the miRNA content between EVs from static and flow conditions, we did find an increased abundance of specific miRNAs in the EVs compared to the parental cells. MicroRNA‐122‐5p, has been implicated in various cardiovascular diseases. Studies have shown that miR‐122‐5p is upregulated in patients with both stable and unstable coronary artery disease, suggesting its potential role as a biomarker for plaque instability.^[^ ^92^ ^]^ MiR‐122 has been shown to regulate cardiovascular inflammation, autophagy, apoptosis, oxidative stress and functions as a risk biomarker of cardiovascular diseases,^[^ ^93^ ^]^ while endothelium‐targeted inhibition of miR‐122 improved vascular endothelial function in high‐fat diet‐fed mice.^[^ ^94^ ^]^ Circulating miR‐451a has been reported as potential marker of coronary artery aneurysmal disease,^[^ ^95^ ^]^ and its upregulation could stimulate HUVECs proliferation and apoptosis by directly targeting macrophage migration inhibitory factor (MIF), suggesting miRNA‐451a contribution in regulating atherosclerosis.^[^ ^96^ ^]^ Endothelial‐derived miR‐9 plays a key role in the pathogenesis of diabetic cardiomyopathy and regulates the production of ECM proteins and inflammatory molecules in human cardiac microvascular endothelial cells. Using an EC‐specific miR‐9 transgenic model, it was further demonstrated that EC‐derived miR‐9 regulates cardiac fibrosis.^[^ ^97^ ^]^ Inducing miR‐9 mimics in HUVECs enhanced cell proliferation and angiogenesis while simultaneously reducing apoptosis and inflammation. These effects were mediated through the regulation of the MAPK/ERK and PI3K/AKT/mTOR pathways, supporting our findings on the predicted targets of EV‐enriched miRNAs.^[^ ^98^ ^]^

Our analysis revealed a correlation between miRNA and mRNA levels in cells. Differentially expressed miRNAs are associated with the regulation of their predicted mRNA targets, suggesting that miRNAs influence endothelial gene expression under different culture conditions.

However, we found no correlation between cellular mRNA regulation and EV protein content. As illustrated in Figure S7 (Supporting Information), the upregulation or downregulation of mRNAs in cells does not correspond to the same pattern in EV proteins, indicating that additional factors beyond mRNA expression regulate EV protein content.

Similarly, there was no correlation between EV miRNA content and EV protein composition. EV miRNA profiles remained largely unchanged between flow and static conditions, suggesting that the differences in EV protein composition are not driven by miRNA‐mediated regulation.

Given that intracellular miRNA/mRNA expression correlates but does not translate to EV protein content, we propose that the observed variations in EV protein composition are influenced by selective packaging mechanisms that regulate protein incorporation into EVs,^[^ ^99^, ^100^, ^101^ ^]^ biomechanical stress‐induced changes in cellular signaling affecting EV biogenesis or secretion,^[^ ^102^, ^103^, ^104^ ^]^ and post‐translational modifications^[^ ^105^, ^106^, ^107^, ^108^ ^]^ or differential protein stability independent of transcriptional control.

In summary, we developed an in vitro model for culturing endothelial cells under physiologically relevant conditions, enabling large‐scale EV isolation. We tested various culture media to identify one that minimizes FCS‐derived contaminants while maintaining the stability of primary HUVECs under flow conditions. Our detailed proteome and transcriptome analysis suggested that EV content differs between static and laminar flow cultures, potentially affecting EV‐mediated communication. In this study, we observed significant differences in the expression of specific miRNAs and proteins in EVs produced under varying culture conditions. While these findings provide valuable insights into the impact of culture conditions on EC‐EV composition, the underlying mechanisms driving these changes remain to be fully elucidated.

It is well‐documented that HUVECs exhibit sex‐specific differences in response to mechanical stimulation, gene expression, and other functional characteristics, which could influence EV production, composition, and quality. One limitation of our study is the exclusive use of female HUVECs for the core EV isolation and omics workflows. While this approach was chosen to minimize biological variability and ensure reproducibility, we acknowledge that sex‐specific differences in endothelial function and shear responsiveness have been documented (e.g., in proliferation, apoptosis sensitivity, and transcriptional profiles).^[^ ^109^, ^110^, ^111^, ^112^ ^]^ Although these differences are often quantitative rather than qualitative in nature, future studies should incorporate matched male and female donors to systematically investigate sex‐dependent aspects of EV release and content. The method we have established provides a foundation for conducting sex‐specific studies, as well as investigations into drug effects, different shear stress conditions (e.g., intensity, oscillatory flow), the impact of hormones, and other physiological factors. These future directions will contribute to a deeper understanding of the biological and functional heterogeneity of EVs across various experimental conditions.

## Conflict of Interest

The authors declare no conflict of interest.

## Supporting information



Supporting Information

Supplemental Table 1

Supplemental Table 2

Supplemental Table 3

## Data Availability

The data that support the findings of this study are available from the corresponding author upon reasonable request.
