# Supplementary material for: Laminar Flow Alters EV Composition in HUVECs: A Study of Culture Medium Optimization and Molecular Profiling of Vesicle Cargo
Source: Small Methods. 2025 Jul 4;9(8):2401841. doi: 10.1002/smtd.202401841 (PMC12391657; doi:10.1002/smtd.202401841)
Supplement: Supplementary file 1 — Supporting Information [file SMTD-9-2401841-s001.docx]

Supporting Information

**Laminar Flow Alters EV Composition in HUVECs: A Study of Culture Medium Optimization and Molecular Profiling of Vesicle Cargo**

*Arefeh Kardani^1^, Jan Hemmer^1^, Britta Diesel^1^, Vida Mashayekhi^1^, Annika Schomisch^1^, Marcus Koch^2^, Claudia Fecher-Trost^3^, Markus R Meyer^3^, Nicole Ludwig^4,5^, Shusruto Rishik^5^, Andreas Keller ^5^, Jessica Hoppstädter^1^, Gregor Fuhrmann,^6,7*^, Alexandra K. ​​Kiemer^1,8,9*^*

^1^Saarland University, Department of Pharmacy, Pharmaceutical Biology Campus C2 3, 66123 Saarbrücken, Germany

^2^INM - Leibniz Institute for New Materials, Campus D2 2, 66123 Saarbrücken, Germany

^3^Department of Experimental and Clinical Toxicology, Institute of Experimental and Clinical Pharmacology and Toxicology, Center for Molecular Signaling (PZMS), Saarland University, Kirrberger Str., Building 46, 66421, Homburg, Germany

^4^Core Facility Molecular Single Cell and Particle Analysis, Medical Faculty, Saarland University, Homburg, Germany

^5^Chair for Clinical Bioinformatics, Saarland Informatics Campus, Saarland University, Saarbrücken, Germany

^6^Friedrich-Alexander-University Erlangen-Nürnberg, Department of Biology, Pharmaceutical Biology, Staudtstr. 5, 91058 Erlangen, Germany

^7^FAU NeW – Research Center New Bioactive Compounds, Nikolaus-Fiebiger-Str. 10, 91058 Erlangen, Germany

^8^PharmaScienceHub (PSH), Saarland University, Saarbrücken, Germany

^9^Center for Gender-Specific Biology and Medicine (CGBM), Saarland University, Germany

*Corresponding author, phone: +49 681 302 57301 (AKK), email: pharm.bio.kiemer@uni-saarland.de,

**Figure S1.** Volcano plot of differentially expressed genes (DEGs) between flow and static conditions. Red and blue dots represent significantly upregulated (759) and downregulated (608) genes under flow conditions, respectively (adjusted p-value < 0.05, fold change > 2). Genes included in the KEGG pathway “Fluid Shear Stress and Atherosclerosis” are highlighted.

**
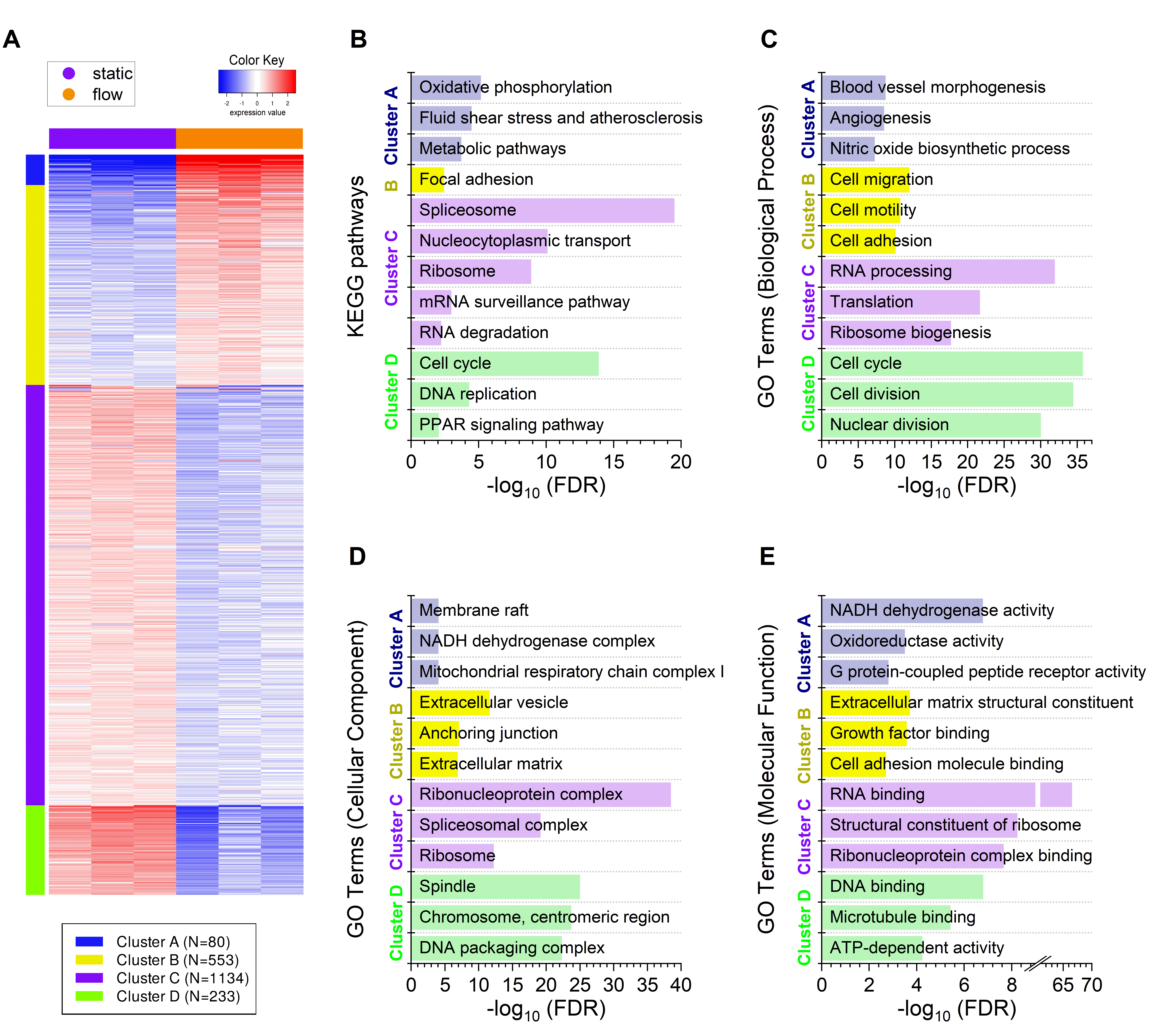
**

**Figure S2.** Clustering and pathway enrichment of the 2,000 most variable genes.

(A) Heatmap of the 2,000 most variable genes, clustered using k-means clustering. Expression levels are displayed as log10-transformed RPKM values, with genes centered by subtracting the mean expression across samples. The four identified clusters are color-coded: Cluster A (blue), Cluster B (yellow), Cluster C (violet), and Cluster D (green).

(B–E) Pathway enrichment analysis for each cluster. Selected pathways are shown; see Supplemental Table 2 for the full list.

(B) KEGG pathway enrichment analysis.

(C) GO Biological Process enrichment analysis.

(D) GO Cellular Component enrichment analysis.

(E) GO Molecular Function enrichment analysis.

**
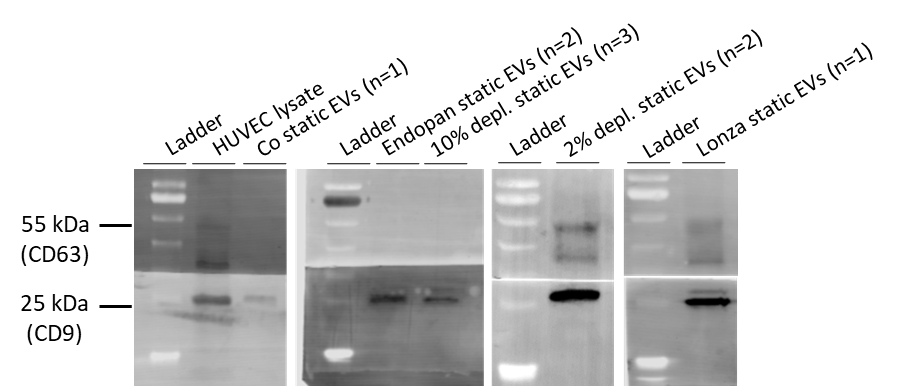
**


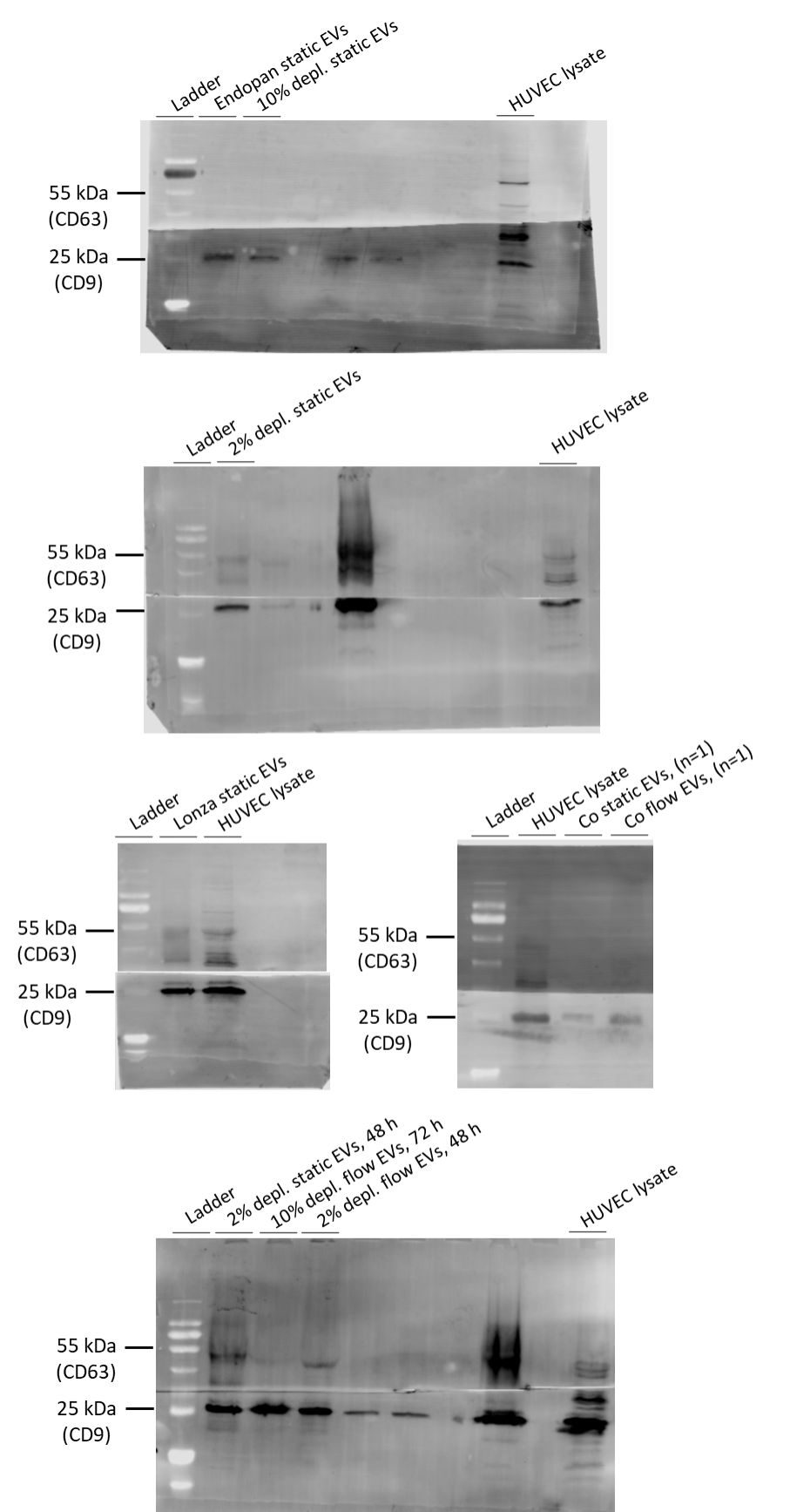


**Figure S3.** Western blot analysis of HUVEC EVs isolated form static cultures in different media after 72 h. Cells were a mix of two HUVEC donors with unknown sex. Number of biological replicates is shown in parenthesis. Uncut western blots of HUVEC EVs from static and flow cultures (20 dynes cm^-2^).


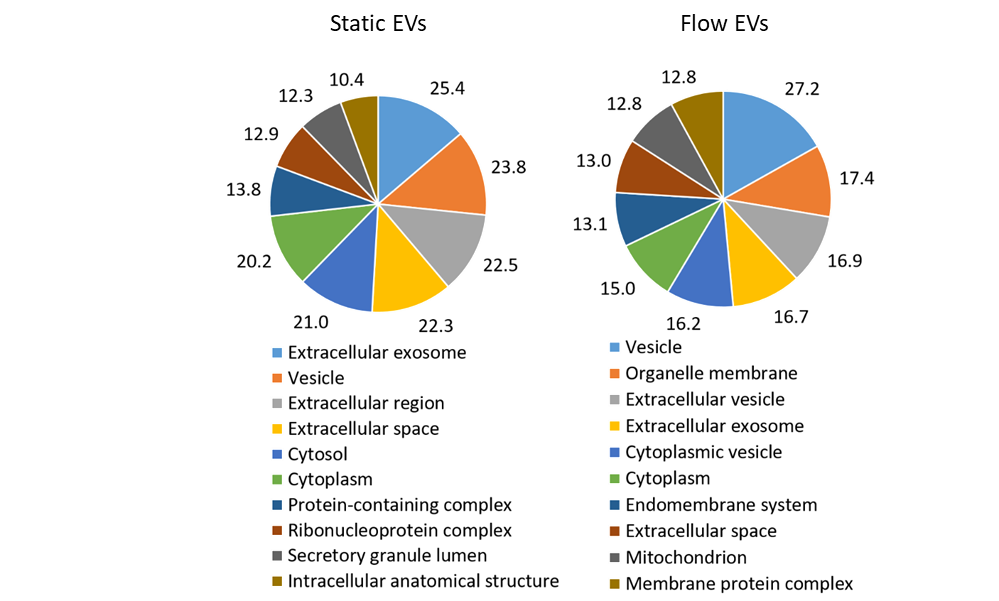


**Figure S4.** Top 10 gene ontology (GO) cellular component terms for significantly enriched proteins in static and flow EVs according to the STRING database. –Log_10_ (p-value) is shown for each term.


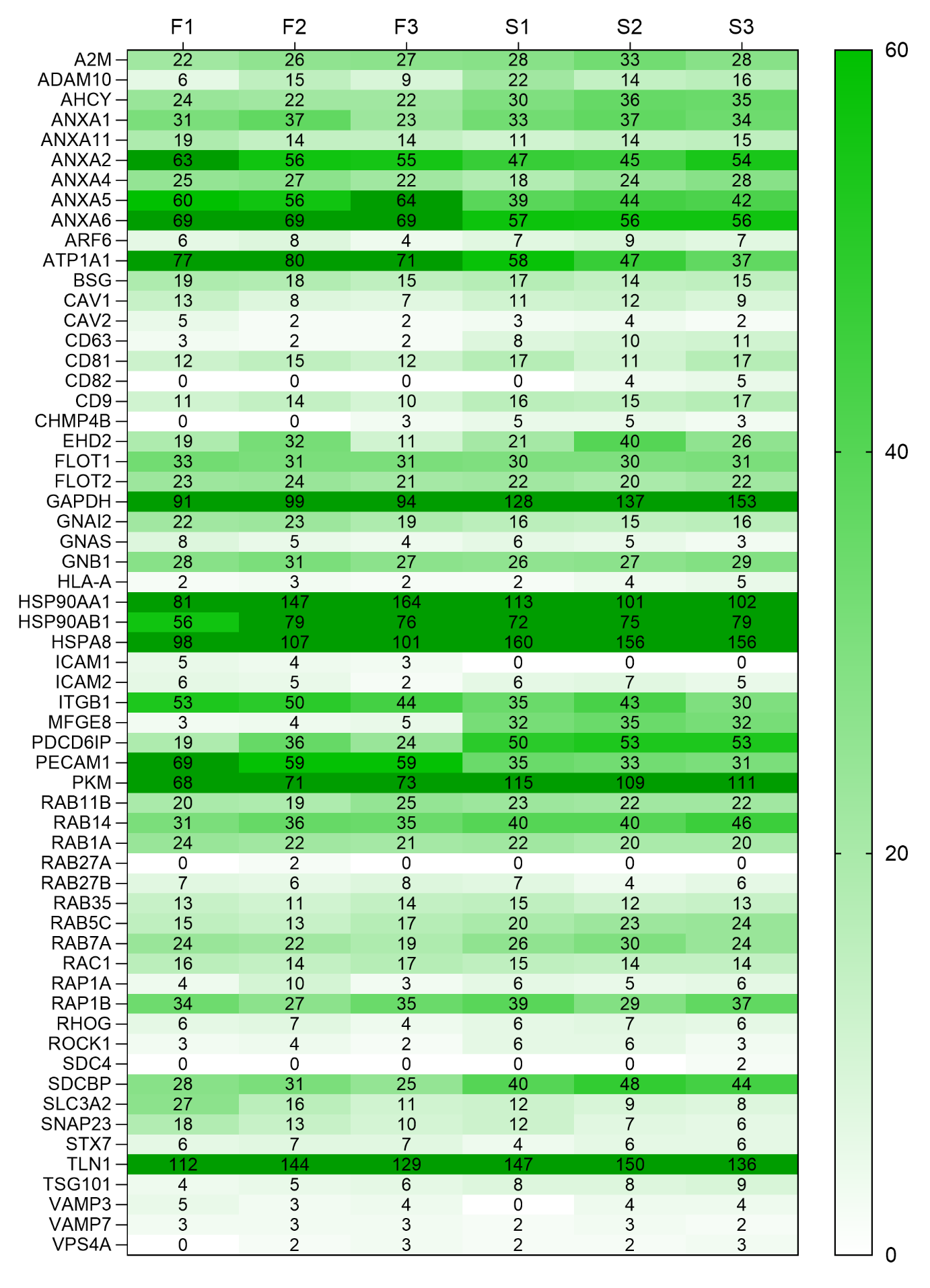
**Figure S5**. EV marker distribution in static and flow EVs. Exclusive unique spectrum count raw data are shown for all three independent preparations per condition (S: static EVs, F: flow EVs). N= three biological replicates, each replicate is a mix of three HUVEC female donors.

|  | #cells by CM collection | Volume of CM | Protein concentration (BCA) of unpurified EV pellet (after UC) | Protein concentration (BCA) of purified EVs (SEC + VIVASPIN 500 5KD MWCO for concentration) | Total purified EV volume | Total protein of purified EVs |
| --- | --- | --- | --- | --- | --- | --- |
| Test 1 | 20 Mio | ~65 ml | 2 mg/ml  (total volume: 150 µl) | 0.016 mg/ml | 50 µl | 0.8 µg |
| Test 2 | 20 Mio | ~65 ml | 3 mg/ml  (total volume: 150 µl) | 0.45 mg/ml | 50 µl | 22.5 µg |

**Figure S6.** Protein concentration of HUVEC EVs collected via size exclusion chromatography (SEC) and concentration Step. HUVEC EVs were isolated using ultracentrifugation (UC). The pellet was resuspended in 150 µl of buffer. Protein concentration of the EVs with was measured with BCA assay. The EVs were then loaded onto a size exclusion chromatography (SEC) column, and 20 fractions of 500 µl each were collected. A BCA assay was performed on each fraction to determine its protein content. Based on BCA results, fractions 1–7 were pooled and further concentrated using a VIVASPIN 500 (5 kDa MWCO) column.


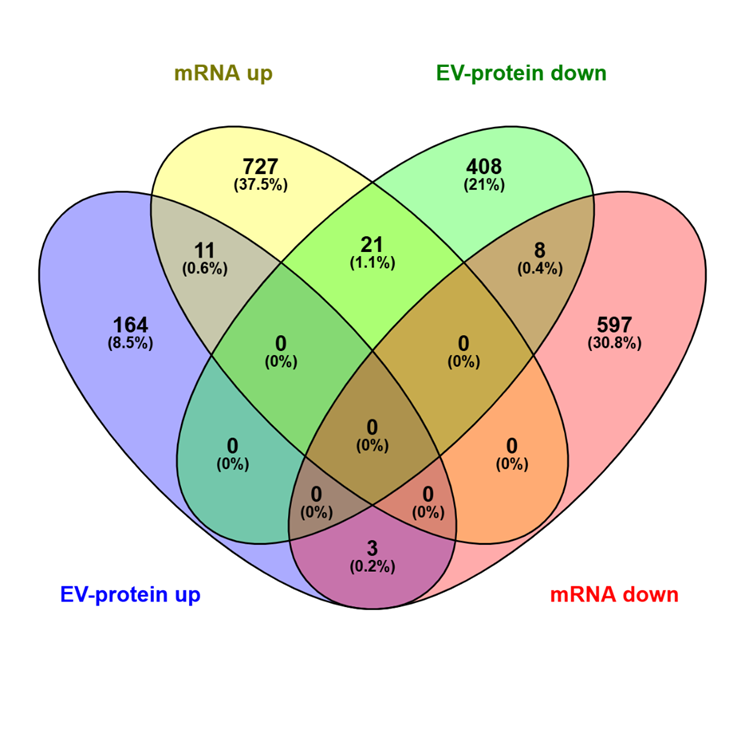

**Figure S7.** Overlap between differentially expressed mRNAs and EV proteins.

The Venn diagram shows the distribution of differentially expressed mRNAs in cells (mRNA up, yellow; mRNA down, red) and differentially expressed proteins in EVs (EV-protein up, blue; EV-protein down, green). Numbers indicate the count of overlapping and unique entities in each category, with percentages representing their proportion relative to the total.
